# Spatio-Temporal Metabolite and Elemental Profiling of Salt Stressed Barley Seeds During Initial Stages of Germination by MALDI-MSI and µ-XRF Spectrometry

**DOI:** 10.3389/fpls.2019.01139

**Published:** 2019-09-25

**Authors:** Sneha Gupta, Thusitha Rupasinghe, Damien L. Callahan, Siria H. A. Natera, Penelope M. C. Smith, Camilla B. Hill, Ute Roessner, Berin A. Boughton

**Affiliations:** ^1^School of BioSciences, University of Melbourne, Parkville, VIC, Australia; ^2^Metabolomics Australia, School of BioSciences, University of Melbourne, Parkville, VIC, Australia; ^3^School of Life and Environmental Sciences, Deakin University, Burwood, VIC, Australia; ^4^AgriBio, Centre for AgriBiosciences, Department of Animal, Plant and Soil Sciences, School of Life Sciences, La Trobe University, Bundoora, VIC, Australia; ^5^School of Veterinary and Life Sciences, Murdoch University, Murdoch, WA, Australia

**Keywords:** salinity, barley, germination, metabolomics, mass spectrometry imaging, lipids, MALDI

## Abstract

Seed germination is the essential first step in crop establishment, and can be severely affected by salinity stress which can inhibit essential metabolic processes during the germination process. Salt stress during seed germination can trigger lipid-dependent signalling cascades that activate plant adaptation processes, lead to changes in membrane fluidity to help resist the stress, and cause secondary metabolite responses due to increased oxidative stress. In germinating barley (*Hordeum vulgare*), knowledge of the changes in spatial distribution of lipids and other small molecules at a cellular level in response to salt stress is limited. In this study, mass spectrometry imaging (MSI), liquid chromatography quadrupole time-of-flight mass spectrometry (LC-QToF-MS), inductively coupled plasma mass spectrometry (ICP-MS), and X-ray fluorescence (XRF) were used to determine the spatial distribution of metabolites, lipids and a range of elements, such as K^+^ and Na^+^, in seeds of two barley genotypes with contrasting germination phenology (Australian barley varieties Mundah and Keel). We detected and tentatively identified more than 200 lipid species belonging to seven major lipid classes (fatty acyls, glycerolipids, glycerophospholipids, sphingolipids, prenol lipids, sterol lipids, and polyketides) that differed in their spatial distribution based on genotype (Mundah or Keel), time post-imbibition (0 to 72 h), or treatment (control or salt). We found a tentative flavonoid was discriminant in post-imbibed Mundah embryos under saline conditions, and a delayed flavonoid response in Keel relative to Mundah. We further employed MSI-MS/MS and LC-QToF-MS/MS to explore the identity of the discriminant flavonoid and study the temporal pattern in five additional barley genotypes. ICP-MS was used to quantify the elemental composition of both Mundah and Keel seeds, showing a significant increase in Na^+^ in salt treated samples. Spatial mapping of elements using µ-XRF localized the elements within the seeds. This study integrates data obtained from three mass spectrometry platforms together with µ-XRF to yield information on the localization of lipids, metabolites and elements improving our understanding of the germination process under salt stress at a molecular level.

## Introduction

Barley is a model organism for investigating the cereal germination process (Gorzolka et al., 2016) which commences with the uptake of water by the quiescent dry seed and finishes with the emergence of the radicle through the seed coat. Seed germination requires sufficient moisture and is also affected by temperature. Salinity affects normal seed germination through osmotic stress (Bliss et al., 1986), ion toxicity (Hampson and Simpson, 1990), or a combination of both (Huang and Steveninck, 1988). High intracellular concentrations of both Na^+^ and Cl^−^ can inhibit the metabolism of dividing and expanding cells (Neumann, 1997), restrict mobilization, and hinder seedling emergence (Marques et al., 2013; Alencar et al., 2015). These conditions result in retarded (Zhang et al., 2010) or delayed (Ashraf et al., 2003) germination. Barley genotypes can be classified as either tolerant or sensitive to salt stress depending upon their genetic diversity and the ability to germinate and, survive under these conditions (Shelden and Roessner, 2013; Shelden et al., 2013; Shelden et al., 2016). The molecular changes in barley seed during germination that may play a role in tolerance or sensitivity are important in determining a plant’s overall response to salinity.

Mature seeds contain a variety of compounds such as proteins, carbohydrates, lipids, vitamins, and phenolics. These compounds provide essential nutrients for the seed to germinate and develop into a mature plant, and are synthesized, packaged and stored in specific tissues that are chemically and morphologically distinct from each other (Fulcher, 1982). For example, a reserve of carbohydrates (starch) is found in the endosperm of cereal seed whereas a reserve of lipids and proteins is found in the scutellum.

Lipids are vital and abundant cellular constituents responsible for the structure and function of cell membranes, and act as an energy store to allow metabolism to continue during abiotic stress. Recent research has shown that lipids are involved as signal mediators in the initiation of defence reactions (Sarabia et al., 2018). Lipids are also involved in processes to mitigate the effects of stress in plant cells (Hasanuzzaman et al., 2013; Okazaki and Saito, 2014) for example through remodelling of glycerolipid levels to maintain membrane integrity and optimal fluidity (Sarabia et al., 2018). Plants also produce a vast array of secondary metabolites such as carotenoids, flavonoids, and coumestans (Gry et al., 2007). These compounds have long been known to protect plants from different biotic and abiotic stresses, function as signal molecules and act as antimicrobial agents (Bartwal et al., 2013).

Although changes in profiles of protein (Yang et al., 2007), transcripts (Nakabayashi et al., 2005; Reyes and Chua, 2007), and metabolites (Fait et al., 2006; Howell et al., 2009) during germination are well documented, there is currently little information on molecular changes during seed germination in response to abiotic stress including salinity. With the current changes in the global climate (Karl et al., 2009), understanding these responses to stress will be more important than ever. Information on how an embryo mobilizes its internal reserves during the early stages of germination in saline conditions can provide insights into the metabolic process of germination; and consequently help us understand how certain crop genotypes germinate better under adverse environmental conditions (Achakzai, 2014).

To understand the role and function of metabolites and lipids within an organism at the tissue level, spatial information about target compounds is required (Li et al., 2008). Many studies analyze whole tissue responses (Fiehn, 2002; Sumner et al., 2003; Weckwerth, 2003; Cevallos-Cevallos et al., 2009; Lee et al., 2010), and so information about the localization of small molecules in cell types within the tissue is lost.

Mass Spectrometry Imaging (MSI) provides a platform to detect and localize intra-tissue variation and the spatial distribution of metabolites at the cellular and sub-cellular levels at high resolution (Lee et al., 2012; Boughton et al., 2016). MSI enables visualization of the distribution of individual biomolecules in a tissue section without requiring staining or complicated pretreatment (Cornett et al., 2007). This method of direct analysis of a tissue section (Zaima et al., 2009) allows detection of a wide range of endogenous molecules such as lipids, peptides, and secondary metabolites (Boughton et al., 2016; Hansen et al., 2018; Heskes et al., 2018). In plants, MSI has been used to target small molecules in different plant species and tissues. Recent examples include *Arabidopsis* leaves (Shroff et al., 2008), Medicago roots (Ye et al., 2013), wheat seeds and stems (Burrell et al., 2006; Robinson et al., 2007), soya leaves (Mullen et al., 2005), rice seeds (Zaima et al., 2010a), different tissues of Eucalyptus species (Hansen et al., 2018; Jakobsen Neilson et al., 2019), and barley roots (Sarabia et al., 2018). Novel metabolites were discovered using Matrix Assisted Laser Desorption Ionization-Mass Spectrometry Imaging (MALDI-MSI) (Gorzolka et al., 2014), further highlighting the benefit of obtaining spatially resolved information.

In this study, we used a combination of four high-end analytical techniques — MALDI-MSI, liquid chromatography-tandem mass Spectrometry (LC-QToF-MS/MS), inductively coupled plasma mass spectrometry (ICP-MS), and micro X-ray fluorescence (µ-XRF), to give new insights into the metabolism of metabolites including lipids, as well as elements including Na^+^ and K^+^. These analytical platforms were used to analyze early stages of germination in two barley genotypes that differ in their germination phenology (Mundah-early germinating and Keel-late germinating) under salt stress. Barley was chosen as it is not only an agriculturally and industrially important crop, but also exhibits a typical cereal germination process, allowing the results to be applied to other cereal crops.

This combined approach provides unique insights into the spatially resolved coordination of metabolic processes that are sequestered among the germinating embryo axis, the lipid rich scutellum, the nutritive endosperm, the digestive enzyme secreting aleurone and the outer pericarp cell layers during the early stages of seed germination. LC-QToF-MS provided information on the distribution of a specific flavonoid in five additional barley genotypes to determine potential roles in response to salt stress. On-tissue analysis using MSI-MS/MS further confirmed the identity of a tentative flavonoid resulting in nine fragment ions. Techniques such as ICP-MS and µ-XRF were used to provide data on element levels that are dynamically mobilized during barley seed germination.

## Material and Methods

### Plant Material

Seven varieties of barley were selected based on their importance for crop production, commercially relevant traits, and known difference in germination phenology and salinity tolerance (Tavakkoli et al., 2012; Kamboj et al., 2015; Sarabia et al., 2018; Yu et al., 2018). This selection comprised of two Australian barley feed (Mundah and Keel), one Australian food (Hindmarsh), and three malting cultivars (Gairdner, Vlamingh, and Clipper), as well as one North African landrace (Sahara). All seeds were sourced from The University of Adelaide, Australia.

### Seed Germination and Sample Collection

The germination of seven barley varieties was compared under normal and saline conditions. Twenty seeds from each variety were sterilized in 70% ethanol for 1 min. They were then rinsed 4–5 times in sterile 18.2 Ω deionized water before being treated with 1.0% (v/v) bleach for 10 min, followed by thorough washing in sterile 18.2 Ω deionized water 6–7 times. Seeds were then imbibed overnight (∼16 h) in sterile 18.2 Ω deionized water with continuous aeration before being transferred to 1% agar plates containing a range of salt (NaCl) concentrations: 0 (control), 50, 100, 125, 150, 200, and 250 mM. The plates were then sealed with parafilm, wrapped in aluminium foil and kept at 4°C for 48 h before being transferred to a growth chamber maintained at 17°C constant temperature with no light. Plates were scored for the number of seeds germinating at different times after transfer to the growth chamber (48, 72, 96, and 120 h post-imbibition). The germination percentage was calculated using the following equation: Germination (%) = G/X*100, where, G = number of seeds germinated and X = number of seeds sown.

Seeds from barley varieties with the highest and lowest germination frequencies under salinity, respectively, were used in a second experiment to produce germinating seeds for MALDI-MSI, ICP-MS, and µ-XRF. The seeds were sterilized as described above. After 16 h of overnight aeration, seeds were harvested for the zero hour time point (0 h) and the remaining seeds were aseptically transferred to petri dishes with and without saline agar. These dishes were kept in the dark at 4°C for 48 h and then transferred into a growth chamber maintained at a constant temperature of 17°C with no light. Germinating seeds were harvested at 8, 16, 24, 48, or 72 h growth during this time so that seeds at different developmental stages could be analyzed. These seeds were immediately frozen in Super Cryo Embedding Medium (SCEM, Section Lab) using liquid nitrogen and stored at −80°C into 50 ml Conical Centrifuge Tubes (Thermo Fisher Scientific, USA) until subjected to cryosectioning.

### Chemicals

Solvents were purchased from Merck Millipore (Bayswater, VIC, Australia), Chemicals including 2,5-dihydroxy benzoic acid (DHB), elemental red phosphorus and Supra pure® nitric acid (70%) were purchased from Sigma-Aldrich (Castle Hill, NSW, Australia). Embedding and freezing supplies including a cryofilm fitting tool set (2.0 cm), embedding container (1.5cm × 2.0 cm), embedding medium (SCEM), and cryofilm 2C(9) (2.0 cm in width), were purchased from Section-Lab Co. Ltd. (Tokyo, Japan). Sectioning supplies including Menzel-Gläser Superfrost Ultra Plus Glass slides, Optimal Cutting Temperature (O.C.T.) compound and Feather® C35 tungsten microtome blades were purchased from Grale HDS (Ringwood, Australia). Lysing Matrix Tubes with 0.5 g Lysing Matrix D (1.4 mm ceramic spheres) were purchased from MP Biomedicals (Seven Hills, NSW, Australia). Elemental standards were purchased from PerkinElmer (Melbourne, VIC, Australia). Aqua Regia (concentrated hydrochloric acid and concentrated nitric acid in the ratio 4:1) was prepared fresh immediately prior to use.

### MALDI-MSI: Sample Preparation and Imaging

For MALDI-MSI, samples from six time points during germination (**Supplementary Figure S1**) of the two barley varieties were analyzed. Prior to sectioning, frozen seed samples were kept in a Reichert Jung 2800 FRIGOCUT M cryostat at −20°C for 30 min. Later, the frozen sample was attached to a cold specimen disk using Optimal Cutting Temperature (OCT) compound and trimmed with a C35 tungsten microtome blade (Feather®). Longitudinal sections of 14 µm were stabilized with cryofilm 2C(9) (Section Lab, Japan), attached to a glass slide with electrically conducting double-sided tape according to the Kawamoto’s film method with slight modifications (Kawamoto, 2003; Zaima et al., 2010b; Boughton et al., 2016), and immediately freeze dried in a Christ Alpha 1–4 LD plus Freeze Drier (John Morris Scientific, Australia) under vacuum (1.0 mbar) for 20 min. This step was important in order to eliminate water from seed sections and also to facilitate their manipulation and storage at room temperature before further analysis. The sections were chosen from the centre of the seed so the embryo, endosperm and aleurone layer were included in all sections. In order to account for biological variation present, at least three different seeds from each time point and treatment were analyzed. These were treated as technical and biological replicates.

2,5-dihydroxy benzoic acid (DHB) was used as sublimation matrix for positive mode analyses. For this study, the optimal conditions were 0.11 Torr pressure during sublimation, a hot plate temperature of 140°C, 300 mg DHB as matrix added to the sublimator, and a sublimation period of 4 min.

MS imaging of seed sections was performed on a Bruker SolariX 7T hybrid ESI-MALDI-FT-ICR mass spectrometer equipped with a Smartbeam II laser (Nd : YAG laser 355 nm) operating at 2 kHz, using the “minimum” focus setting and smart walk enabled (Bruker, Bremen, Germany). For every run, laser fluence was optimized to obtain the best signal-to-noise (S/N) ratio. The MS was operated in the positive ionization mode across the mass range *m/z* 150–2,800. MSI measurements were acquired with a raster of 75 × 75 µm. The measurement of the spots was carried out in random order to eliminate influences of measurement order. An external calibration (quadratic equation) was carried out using elemental red phosphorus obtaining a minimum of seven points of calibration over the mass range. A mass accuracy better than 4 ppm was obtained across a tissue section image. Co-registration between each MSI data set and its optical image was performed with flexImaging software v.4.1 (Bruker Daltonics, Bremen, Germany).

MS/MS spectra of selected ions, previously identified through SCiLS Lab (SCiLS Lab 2017a, SCiLS GmbH) analysis were conducted in positive mode using a laser power of 40%. A total of 5,000 laser shots per sample were fired randomly across the relevant tissue region. The isolation window was set to 5 *m/z* with the collision RF amplitude set to 1,200.0 Vpp and collision voltage set to 20 V.

### MALDI-MSI: Analysis, Data Processing, Detection and Annotations

The raw MALDI-MSI datasets were analyzed using flexImaging software v.4.1 (Bruker Daltonics, Bremen, Germany) and normalized to the Root Mean Square (RMS). Any *m/z* peaks that showed spatial distribution across the tissue sections were manually annotated and used to create a list of spatially distributed potential mass features. The generated list was further analyzed to identify protonated ([M+H]^+^), sodiated ([M+Na]^+^) and potassiated ([M+K]^+^) adducts of the mass features that displayed spatial distribution prior to identification of potential metabolites using accurate mass search against the LIPID MAPS (Fahy et al., 2007) databases by accurate precursor mass (<5 ppm). Further average MS spectra were also exported from flexImaging and imported into SimLipid v.5.50 (Premier Biosoft International, Palo Alto, CA, USA) for accurate mass annotation. Tentative annotations were performed to level two based on accurate mass match and spectral libraries (Sumner et al., 2007). All potential lipid and metabolites detected with multiple adducts were collapsed into single ID for identification and analysis purposes.

Annotated *m/z* features were imported into SCiLS Lab (SCiLS Lab 2017a, SCiLS GmbH; (http://scils.de/; Bremen, Germany) software from flexImaging and further used to identify discriminant features between control sections and treatments. Sub regions were created to analyze discriminant lipids in embryo and endosperm regions. Normalization of data was performed according to the Total Ion Count (TIC) of all spectra prior to data analysis. Next, discriminative *m/z* values were identified from all individual spectra with a minimal interval width of ±5 mDa between control and salt-treated sections. A computer-generated diagram known as a Receiver Operating Characteristic (ROC) curve was used to quantify how well the *m/z* values discriminated between two treatments (control and salt). Strict ROC Area Under the Curve (AUC) values of ≥0.8 and ≤0.2 were set as an additional criterion to the Student’s *t*-test *p*-value of <0.005 for peaks considered as statistically significant for salt vs control and control vs salt respectively (Rauser et al., 2010). The Intensity Box Plot chart was generated which depicts intensities of a given *m/z* interval filtered by the visible regions through their quartiles.

Peak lists were then exported and reimported into flexImaging to verify and confirm the spatial distribution of identified discriminant peaks. After analysis, a curated list of spatially distributed peaks was generated for control vs salt-treated images. For MS/MS analysis, raw data was analyzed on Bruker Compass DataAnalysis v5.0 using the deconvolution and manual annotation tools.

### ICP-MS: Sample Preparation and Analysis

Dry seeds were ground to a fine powder using a Tube Mill control (IKA Mills, Selangor, Malaysia) at 25,000 rpm (30,500 × g) for 4 min. The ground samples were accurately weighed (20 mg) into 10 ml Falcon tubes (Sarstedt, Australia). These samples were then digested in 300 µl acid solution (Aqua Regia) by heating for 1.5 h at 80°C. After digestion and cooling to room temperature, deionized water was added to make the final volume to 10 ml. The tubes were then centrifuged at 5,000 rpm (6,100 × g) for 5 min. The supernatant was used for ICP-MS analysis.

A modified method from Callahan et al. (2016) was used for this study. An ICP-MS instrument (NexION 350X, PerkinElmer, USA) was used to measure the concentration of the following elements: P, S, K, Ca, Mg, Mn, Co, Ni, Cu, Zn, and Fe. The internal standards Sc (200 ppb) and Rh (20 ppb) in 1% Aqua Regia were used for correction. The internal standard was mixed with the sample stream *via* a T-piece in a 1:1 ratio giving a final acid concentration of 2% at the source. Two sets of calibration standards were prepared. Set 1 contained P, Na, K, and Ca at 500, 1,000, and 5,000 ppb. The second set contained the other elements at 0.1, 1, 10, 50, 100, and 500 ppb. The mass spectrometer was operated in kinetic energy discrimination mode (KED) with 50 ms dwell times, 20 sweeps, one reading and three replicates. The plasma source conditions were: nebulizer gas flow 1.02 L min^−1^, auxiliary gas flow 1.2 L min^−1^, plasma gas flow 15 L min^−1^, ICP RF power 1,500 W.

Data were processed using Syngistix (PerkinElmer) software. Signal responses were normalized to the rhodium (Rh) internal standard, sample weights, and dilution factors.

### µ-X-ray Fluorescence Analysis

An M4 Tornado µ-XRF spectrometer (Bruker, Bremen, Germany) equipped with a Rh anode side window X-ray tube with a polycapillary lens was used to obtain elemental distribution maps in this study. The step size was set to 40 µm (for whole seed) combined with high excitation intensity. Direct analysis on seed sections prepared similarly as described in Section 3.4 (without matrix application). This was achieved by placing the sections on the µ-XRF platform under 20 mbar vacuum, 50 kV voltage, and an anode current of 199 µA conditions (Sarabia et al., 2018). Detailed summary on the instrumental conditions and operating parameters used to analyze barley seeds in this study is given in **Table S1**. Spectra acquisition and 2D elemental maps were obtained using the Esprit software (Bruker, Berlin, Germany).

### LC-QToF-MS: Sample Preparation and Analysis for Flavonoid Detection

Seeds were germinated and harvested as described above (Section 2.2) followed by immediate freeze drying in a Christ Alpha 1–4 LD plus Freeze Drier (John Morris Scientific, Australia) under vacuum (1.0 mbar) for 48 h. This facilitated fine powdering of seeds prior to extraction. A modified method from (Routaboul et al., 2006) was used for extracting flavonoids. Briefly, flavonoid extracts were obtained from 30 mg dried seed powder. Powder was transferred into cryo-mill tubes along with 1 ml acetonitrile/water (75:25; v/v) containing the following internal standards, ^13^C_6_-Sorbitol (0.02 mg/ml) and ^13^C_5_^15^N–Valine (0.02 mg/ml). The samples were homogenized using a cryomill (Bertin Technologies; 6,000 rpm (7,320 × g) for 3 repetitions at 45 s with a 45 s interval between replicates) at −10°C. The mixture was then sonicated for 20 min followed by centrifugation at 13,000 rpm (15,900 × g) for 10 min at room temperature. The supernatant was separated and transferred into a fresh 2 ml tube. The remaining pellet was further extracted with 1 ml acetonitrile/water (75:25; v/v) on a thermomixer, shaking overnight (500 rpm, 4°C). The solution was centrifuged, and the two extracts were pooled, dried in a SpeedVac (Martin Christ Gefriertrocknungsanlagen GmbH, Germany) without heating.

Dried flavonoid extracts were resuspended in 200 µl acetonitrile/water (75:25; v/v), and 5 µl (for MS analysis) or 10 µl (for MS/MS analysis) aliquots were injected onto a Poroshell 120, HILIC-Z, 2.1 × 100 mm (2.7 µm particle size) column (Agilent Technologies, Santa Clara, CA, USA) at 25°C, and analyzed on a LC system coupled to an Agilent Technologies (Santa Clara, CA, USA) 6545B ESI-QToF-MS. Flavonoids were eluted at 0.250 ml/min over 18 min with a binary gradient of ACN-water (90:10, v/v) containing 20 mM ammonium formate and formic acid and water containing 20 mM ammonium formate and formic acid as described by Routaboul et al. (2006). MS data were acquired with an ESI source in positive ion mode. The MS data presented corresponds to ten pooled biological replicates for each treatment group. MS/MS was performed to obtain the MS2 spectra of the identified compound.

### LC-QToF-MS: Data Processing and Analysis

LC-MS data was processed using MassHunter Qualitative Analysis and MassHunter Quantitative Workstation v.B.08.00 software (Agilent Technologies, Santa Clara, CA, USA) for flavonoid identification. Peak spacing tolerance was set to 0.5 *m/z* with a retention time window of 0.1 s. The acquired data was exported and normalized with sample weight and log transformed. Univariate statistical analysis tests (Student’s *t*-test) was carried out using Minitab software. X-fold changes were determined between control and treated samples of seven genotypes.

## Results

### Overview of Experimental Workflow

**Figure 1:**
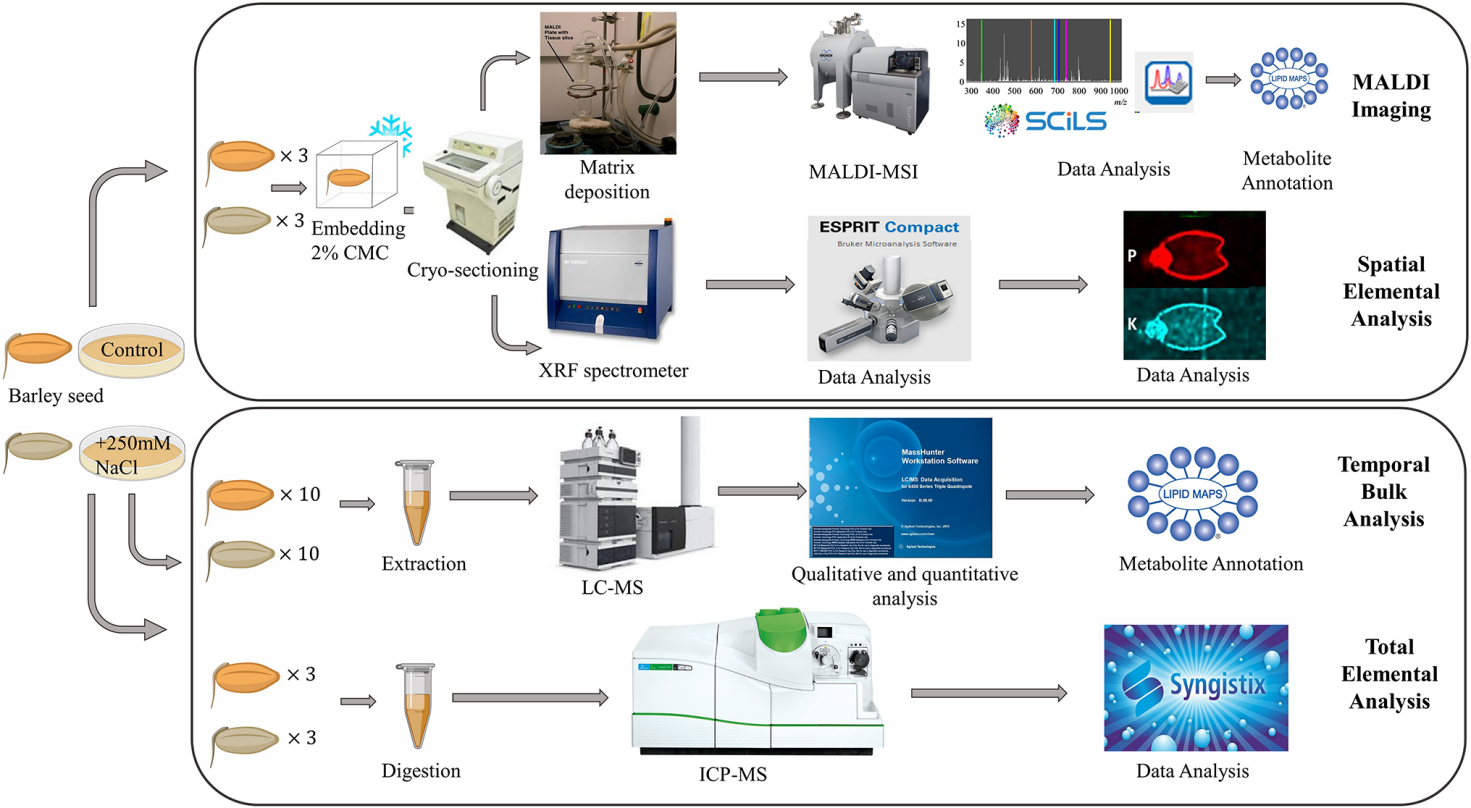
Overall workflow for MALDI-MSI, µ-XRF, ICP-MS and LC-MS analysis of barley seeds. Figure modified from (Sarabia et al., 2018).

A schematic of the experimental workflow is provided in **Figure 1**. MALDI-MSI analysis was performed for seeds grown under control and saline conditions and harvested at six different time points, namely 0, 8, 16, 24, 48, and 72 h post imbibition (**Figure S1**). Sublimation of sectioned tissue with DHB matrix was used for MALDI-MSI positive mode ionization to image the spatial distribution of a wide range of lipids (*m/z* 150–2,800) in the seed sections. ICP-MS (for total elemental analysis) and µ-XRF spectrometry (to obtain the spatial information of targeted elements) was also performed for three time points (0, 24, and 48 h post imbibition).

### Germination Assay: Germination and Seedling Growth Under Salt Stress

In the initial germination assay, seeds from seven barley genotypes (Vlamingh, Mundah, Clipper, Sahara, Hindmarsh, Gairdner, and Keel) were germinated in petri dishes containing different salt concentrations (0–250mM NaCl) and observed for germination from 0–120 h post imbibition (**Table S2**). Seeds were considered to be germinated once emergence of the cotyledon was visible. There was a clear difference in the germination frequency of the seeds germinating in the presence of salt. Keel showed the greatest inhibition (only 65% germinated at highest salt concentration) while all Mundah and Vlamingh seeds germinated. Other genotypes showed germination frequencies between 75 and 90%. The most tolerant genotypes were viable under saline conditions for at least 7 days. Based on this assay, two Australian barley feed varieties were chosen to investigate the metabolic changes that occur during germination under salinity: Mundah, a very early spring variety from Western Australia with very high early vigor (highest germination rate under salinity), and Keel, an early spring variety from South Australia with moderate early vigor (lowest germination rate under salinity) (Long et al., 2003).

### MALDI-MSI of Lipids in Barley Seeds During Germination

**Figure 2:**
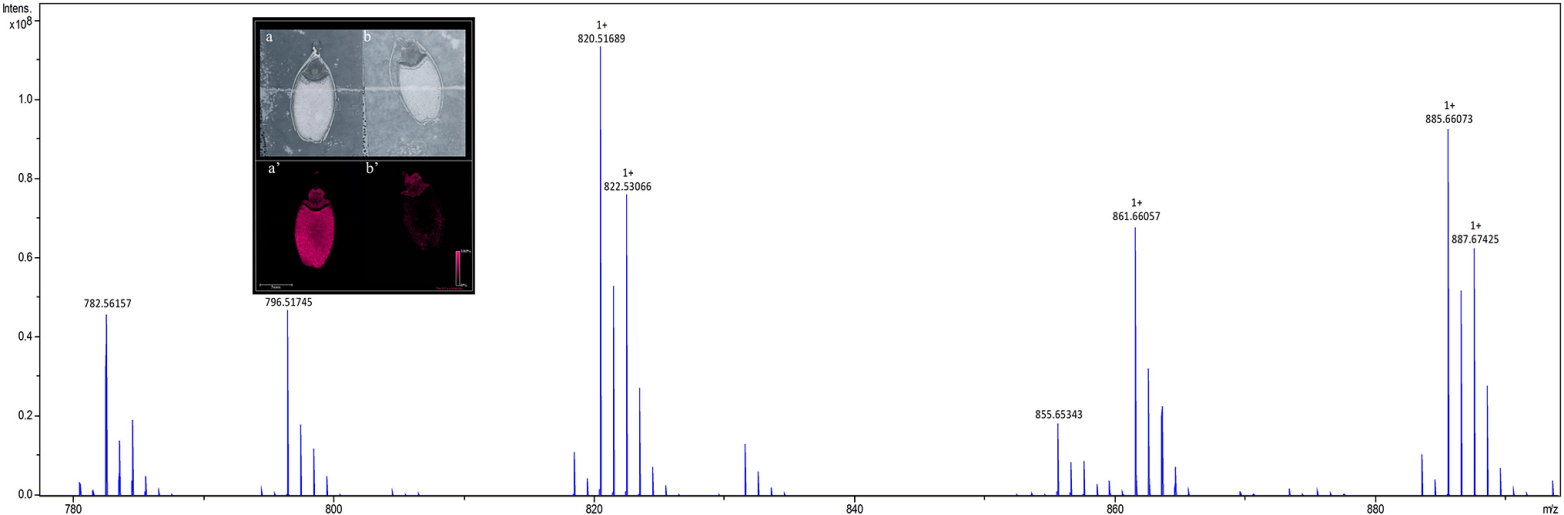
Average mass spectrum between *m/z* 780–890 acquired from barley seed cv. Mundah after 24 h of germination. Each peak illustrates a specific lipid species (or isotope) that can be selected to display the distribution of the lipid across the section. The images obtained at 50 × 50 µm raster size display the different spatial distribution of the lipids across different timepoints, different treatments and between the two genotypes. To ensure the reproducibility of detection of the lipids, three independent seed sections were analyzed for each mass spectrometry imaging experiment. Distribution of the *m/z* 782.5466 PC(36:4) signal in sections of control (a) and salt treated (b) seeds at 24 h are shown in the inset panel. Data was normalized using Root Mean Square.

To identify lipids and other small molecules, the distribution of these compounds at all post imbibition time points in control and saline conditions were compared in both genotypes. Lipids within longitudinal sections of mature barley seeds were profiled by MALDI-MSI at six time points post imbibition (**Figure S1**). Signals derived from the DHB matrix were excluded from further analyses. A total of 774 peaks in the average MS spectra were found to have a differential spatial distribution in the samples. An example of an average mass spectrum between *m/z* 780–900 acquired from a Mundah seed after 24 h post imbibition is presented in **Figure 2**, illustrating a specific peak that can be selected to display the distribution of a corresponding lipid across the section. Many low abundance lipids were not reproducibly detected across all sections due to analytical and biological variations and thus, only the presence and tissue distribution of the tentatively identified lipids observed across three biological replicates are discussed (**Figure S2**).

**Figure 3:**
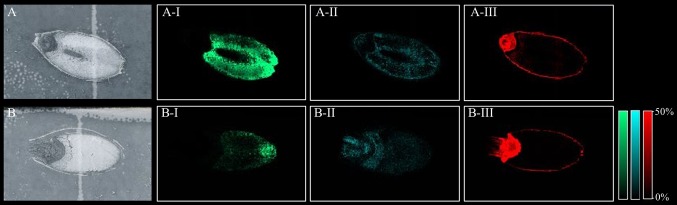
Different lipid species displaying different spatial distribution. **(A)** scanned image of Mundah seed at 48 h in control conditions; **(B)** scanned image of Mundah seed at 48 h in salt conditions; A-I and B-I: *m/z* 496.338 LPC(16:0) showing spatial distribution only in endosperm of both seeds with different intensities in control and salt conditions. A-II and B-II: *m/z* 757.574 PA(40:2) showing spatial distribution in entire seed with different intensities in control and salt treated seed; A-III and B-III: *m/z* 820.530 PC(36:4) showing spatial distribution in the aleurone layer and embryo of control and salt treated seeds. Intensity scale for both images was set to 0-50%. Data was normalized using Root Mean Square.

All peaks showing spatial distribution in the seed sections were selected from flexImaging (**Tables S3 A–D**). Mass precursor ion search (<5 ppm) of the LIPID MAPS database (Fahy et al., 2007) was performed to provide tentative annotations of the features detected and resulted in between 246 to 319 tentatively assigned mass features for each sample. Some of these mass features corresponded to [M+H]^+^, [M+Na]^+^, and [M+K]^+^ ion adducts of the same lipid and were observed in all samples. Two hundred and sixty-nine lipid species were identified in both the Mundah control and salt treated seeds. Three hundred and nineteen lipid species were identified in Keel control seeds but only two hundred and forty-six in salt treated Keel seeds. Lipids were annotated to the sum level only using the Liebisch notation (Liebisch et al., 2013). In-source fragmentation of more complex lipid species, such as Phosphatidylcholine (PC), during detection, may lead to incorrect identification of fragments belonging to other lipid species such as Phosphatidic Acid (PA), Fatty acyls (FA) and Lysophosphatidylcholines (LPC) (Wang, 1999) and this was taken into account during annotation of the lipid species. For example, it was demonstrated that PC, PA, and LPC species had different spatial distribution in seeds (**Figure 3**) suggesting that intact lipid species are being detected and not fragments of other lipids.

Tentatively annotated lipid species with several ion adducts, but the same masses, were collapsed into a single ID. It was noted that the returned matches were filtered to remove tentative annotations from mammalian origins. As a result, 234 lipid species were tentatively identified in Mundah control (**Table S4A**), 202 in salt treated Mundah seeds (**Table S4B**), 155 in Keel control (**Table S4C**) and 145 in salt treated Keel seeds (**Table S4D**). The tentatively annotated lipids were categorized into seven major lipid classes: Fatty acyls (FA), Glycerolipids (GL), Glycerophospholipids (GP), Sphingolipids (SP), Prenol lipids (PR), Sterol lipids (ST), and Polyketides (PK). The percentage of each lipid class in both genotypes is given in **Table 1**. This was solely based on the number of lipid species matched with the LIPID MAPS database. The distribution of lipid classes at all timepoints in both genotypes are shown in **Supplementary Figure S3**. For both genotypes, the number of lipids identified is smaller in the germinated seeds in the presence of salt compared to the control, but in Mundah, the difference between the two treatments is much smaller. The number of lipids identified in each class decreased in Keel after salt treatment, whereas in Mundah, only GL, GP, and ST decreased.

**Table 1:** Percentage of individual lipid classes in two treatments of Mundah and Keel. The values are total percentages of annotated lipids at all timepoints.

	Mundah (%)		Keel (%)	
Lipid classes	Control	Salt	Control	Salt
Fatty acyls	18.70	20.00	26.60	26.62
Glycerolipids	14.07	13.85	24.68	23.55
Glycerophospholipids	49.81	44.92	26.28	34.13
Polyketides	7.96	10.15	7.69	6.48
Prenol lipids	1.85	2.15	5.13	3.07
Saccharolipids	0.56	0.00	0.00	0.00
Sphingolipids	3.89	4.31	3.53	2.39
Sterol lipids	3.15	4.62	6.09	3.75

In Mundah seeds, GP was the major class of lipid altered (43% in Mundah control and 40.6% in Mundah salt). In the GP class, PA, Phosphatidylethanolamine (PE), Phosphoglycerols (PG), and PC were tentatively identified as the major lipid subclasses. The GL class decreased in saline conditions but in control conditions, TAGs were detected in high number. The percentage of ST and GP decreased under salinity, but the sterol/phospholipid ratio was unaffected by salt (0.25% in control and 0.24% in salt).

GP is also the major class altered in Keel seeds (26.3% in Keel control and 34.1% in Keel salt). It is worth noting that an increased number of GP lipids in Keel salt treated seeds was detected likely due to different adducts detected for the same lipid. For example, in the GP class, LPC (16:0), LPC(18:0), and PC(36:5) were found with [M+H]^+^ and [M+K]^+^ ion adducts and PC(34:2) with [M+Na]^+^ and [M+K]^+^ ion adducts. The percentage of ST decreased significantly under salt stress in Keel with a slight decrease in GP under salt stress and hence the sterol/phospholipid ratio was affected by salt in Keel seeds (0.25% in control and 0.12% in salt). It is noted that PC lipid species of 36:n family have been found to be differentially distributed at all timepoints in both genotypes under control and salt conditions.

Gorzolka et al. (2016) performed MSI to localize metabolites during the first seven days of germination in barley seeds and showed the distribution of 11 lipids in barley seeds at different stages of germination and different regions of seeds. In our study, we confirmed the presence of 10 out of 11 lipids from Gorzolka et al. (2016) in Mundah control, 9 in Mundah salt, 4 in Keel control and 9 in Keel salt treated seeds (listed in **Table S5**). Time-dependent changes were also observed in the lipid profile of the endosperm early in germination in Gorzolka et al. (2016). Mono-and diacyl PCs (*m/z* 496, 520, and 758) were highly abundant, and these lipids changed their distribution at later germination stages (Gorzolka et al., 2016). In the current study, the *m/z* 496 and 758 ions could be detected in both genotypes under both treatments. However, they were not consistently observed across all time points. In contrast, the *m/z* 520 ion was only detected in salt treated Keel seeds. The distribution pattern for these three PCs is given in **Table S6**.

### Lipids Detected Immediately After Imbibition and After 8 h of Salt Stress

Comparison between the annotated lipid classes at 0 h and 8 h post-imbibition in control Mundah seed sections showed 26 lipids species present at both time points whereas only 16 lipids species were found in common when 0-hour and 8-hour salt treated seeds were compared (**Tables S7A, B**). In Keel, 30 lipids species were detected in both 0-hour and 8-hour control sections whereas 20 lipids species were found between 0-hour control and 8-hour salt treated seed sections (**Tables S7C, D**). After 8 h of salt stress, 65 lipid species were differentially accumulated compared to the 8 h post imbibition in untreated seeds of Mundah (**Table S8**). The major lipid class detected in 8 h salt treated seeds in Mundah was GP. In contrast, 38 lipids were detected after 8 h of salt stress when compared to the 8 h untreated seeds in Keel (**Table S9**). The major lipid class detected in Keel after exposure to salinity stress was GL.

### Spatial Segmentation of the MALDI-MSI Data Using an Unsupervised Clustering Approach Using SCiLS Lab

Relatively few lipid species changed their spatial distribution in Keel compared to Mundah seeds. In Mundah, out of 225 lipid species that were found to be spatially distributed in salt treated seeds at all time points, 71 (31.5%) were salinity treatment specific. In Keel, out of 163 lipid species spatially distributed in salt seeds at all time points, 48 (29.4%) were specific to salt stress based on LIPID MAPS annotations.

SCiLS Lab was used to determine the discriminative features for both treatments in Mundah and Keel. A univariate measure was used to assess the quality of discrimination for imported *m/z* values for masses created in flexImaging, which quantified how well the *m/z* values discriminated between two treatments. A threshold for Area Under ROC (Receiver Operating Characteristic) Curve (AUC) was used to determine the discriminating masses in both treatments for both genotypes. A perfect discrimination was accepted where AUC value was equal to or close to 1 for salt treated seeds and close to or equal to zero for control seeds. The threshold for this analysis was set to 0.8 for salt treated seeds and 0.2 for control seeds. Subregions were created in SCiLS Lab to find *m/z* values that discriminate between the two treatments for embryo and endosperm in both genotypes. An example of a subregion created for Mundah seeds (*m/z* 615.494) and its corresponding ROC plot for DAG (34:2) with AUC value of 0.087 is given in **Figure S4**. The intensity box plot chart that describes a single *m/z* interval and a single region was also used.

Further discriminant lipids were detected at all timepoints using the above-mentioned threshold values in both genotypes. Two subregions were created — embryo and endosperm. In Mundah embryo, lipids discriminant to control seeds (AUC close to zero) increased in number over time whereas in Keel embryo, the number of discriminant lipids with AUC close to zero decreased over time. In Mundah endosperm, 45 lipids were found to be discriminant between control and salt treated seeds with only nine lipids showing AUC value between 0.8–1.0. In Keel endosperm, only four lipids were discriminant with AUC values between 0–0.2 (**Table S10**).

**Figure 4:**
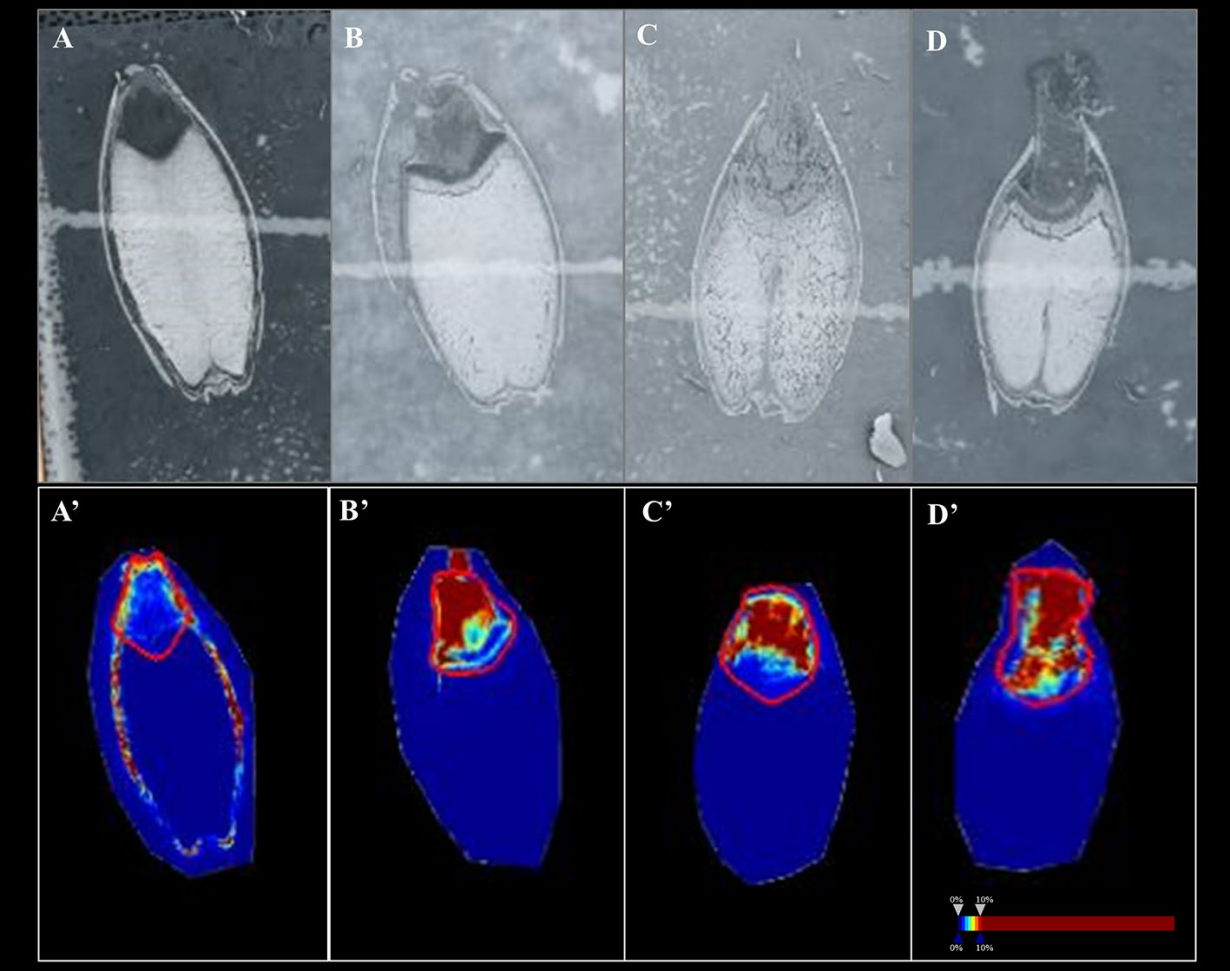
A flavonoid (*m/z* 365.102) with AUC close to 0.8 at 16, 24 and 48 h in Mundah embryos and at 72 h in Keel embryos. **(A, B** and **C)** scanned images of Mundah seeds at 16, 24 and 48 h of salt treatment; **(D)**: scanned image of Keel seed at 72 h of salt treatment; **(A’, B’** and **C’)**: Embryo region showing high intensity of the flavonoid in Mundah salt treated seeds at 24 and 48 h; **(D’)**: Embryo region showing high intensity of the flavonoid in Keel salt treated seed at 72 h. Intensity scale was set to 0–10% for all seeds. Data was normalized using Root Mean Square.

In Mundah embryos, a tentative flavonoid, *m/z* 365.102 [M+H]^+^ (AUC for Mundah 16 h, 0.867; Mundah 24 h, 0.949; Mundah 48 h, 0.907) was found to be strongly discriminant at 16, 24, and 48 h (**Figures 4A–C, A’–C’**) whereas the same mass was observed in Keel embryos at 72 h only (AUC 0.946) (**Figures 4D, D’**). Signal intensity was scaled to optimize visualisation of the respective ion. The intensity box plot and ROC plot for the above mentioned timepoints shows the discrimination capabilities of the given *m/z* for two (control and salt) regions in Mundah at 16, 24, and 48 h (**Figure S5A**) and Keel at 72 h respectively (**Figure S5B**). No common lipids were found in Mundah embryos over all time points. In Keel embryos, diacylglycerols (*m/z* 602.523) and a fatty alcohol (*m/z* 576.508) discriminate between control and salt treatments at 16 and 24 h with an AUC of 0.19 and 0.13 respectively. In Mundah endosperms, it is worth noting that fatty acyls (*m/z* 339.288, [M+Na]^+^) were found in salt treated seeds at 48 h with an AUC 0.88 whereas at 72 h, they were found intensively in control seeds with AUC value of 0.17. No discriminant lipids were found in Keel endosperms. Although several lipid species were found distinct at 8 h salt as compared to 8 h control as mentioned in Section 3.4, no discriminant lipids between these two timepoints in both genotypes were found.

### Confirmation of Tentative Flavonoid Using MS/MS

#### MALDI-MS/MS

**Figure 5:**
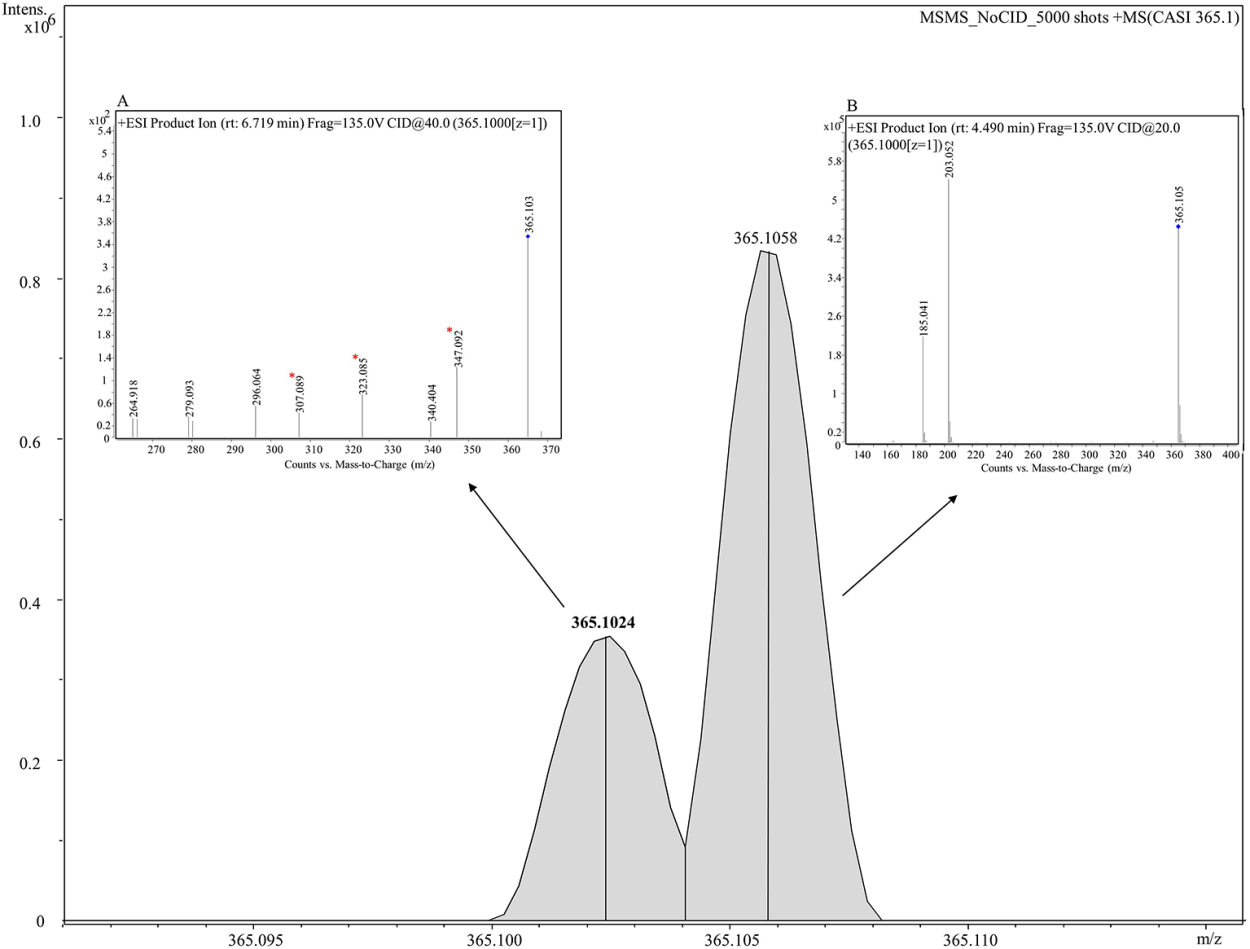
Combined MS and MS/MS spectra of *m/z* 365.1 [M+H]^+^ derived from on tissue MALDI-MS (main figure) and LC-MS/MS (inset figures) respectively. A mass spectrum between 365.090–365.115 is shown in the figure. Two peaks, *m/z* 365.10243 (shown in **bold**) and *m/z* 365.10582 are clearly shown corresponding to a tentative flavonoid and dihexose respectively. Inset **(A)** shows the mass spectrum analyzed using LC-MS/MS for a tentative flavonoid with retention time of 6.719 min giving seven fragments. Three fragments (marked with asterisks) match with fragments obtained from MALDI-MS/MS given in **Figure S6**. Inset **(B)** displays the mass spectrum for a dihexose sugar analyzed using using LC-MS/MS with retention time of 4.49 min and displaying two fragments *m/z* 203.052 and 185.041.

To probe the identity of the tentative flavonoid (*m/z* 365.102 [M+H]^+^), on-tissue MALDI-MS/MS spectra was generated and identified fragments were recorded. A 5 *m/z* window centred around *m/z* 365.1 was isolated with no collision voltage applied. **Figure 5** shows the two distinct peaks with very close mass obtained from MALDI-MS, *m/z* 365.1024 was matched in the LipidMaps and METLIN (http://metlin.scripps.edu) databases to a flavonoid and *m/z* 365.1058 which was assigned as a dihexose sugar (Calvano et al., 2017). Subsequent MS/MS using the same isolation window and application of 20 V collision energy led to observation of nine product ions (**Figure S6**) that were matched with the *in silico* predicted spectra of a flavonoid, Gancaonin F (METLIN database) (Smith et al., 2005), corresponding ions associated with the fragmentation of a dihexose sugar were also observed.

#### LC-MS/MS

LC-MS/MS was performed on seed extracts to provide further evidence for annotation of the possible flavonoid and dihexose sugar. Both MS and MS/MS were conducted with the protonated precursor ion *m/z* 365.1 isolated for MS/MS. The extracted compound chromatogram (ECC) at the MS level showed two separate peaks with retention time of 6.71 and 4.49 min. MS/MS using 40 eV and 20 eV respectively showed distinct differences in the corresponding spectra between the two compounds. Inset **Figure 5A** displays the mass spectrum for one compound with seven fragments, of which three fragments (*m/z* 347, 323, and 307) match closely with *in silico* predicted spectra of Gancaonin F (METLIN database) and MALDI-MS/MS data obtained from on-tissue analysis in this study. Inset **Figure 5B** shows the mass spectrum of a compound with two fragments (*m/z* 203 and 185) that match exactly to dihexose sugar fragments (Calvano et al., 2017). The mass spectrum along with the database search showed a difference of less than 4 ppm in experimental and database mass identified using LC-MS/MS. **Table 2** shows the observed fragments of the tentative flavonoid detected using MALDI-MS/MS and LC-MS/MS that matched with the *in silico* predicted spectra of Gancaonin F.

**Table 2:** Theoretical and observed *m/z* for the tentative flavonoid, Gancaonin F from MALDI-MS/MS and LC-MS/MS.

*In silico* predicted fragments of Gancaonin F	Observed *m/z* by MALDI-MS/MS	MALDI-MSI mass error in ppm	Observed *m/z* by LC-MS/MS	Mass error in Da
347.091	347.092	2.9	347.092	<0.001, <0.001*
337.107	337.103	11.9	–	0.004
335.091	335.092	2.9	–	0.001
333.076	333.076	<0.001	–	<0.001
323.055	323.055	<0.001	323.085	<0.001, 0.03*
321.112	–	–	–	–
309.039	309.040	3.2	–	0.001
307.060	307.060	<0.001	307.089	<0.001, 0.029*
297.039	297.040	3.4	–	0.001
281.044	281.045	3.5	–	0.001

In silico fragments are obtained from METLIN (http://metlin.scripps.edu).

*mass error observed from LC-MS/MS.

### Level of Tentative Flavonoid Increases in Response to Salinity Stress in Four Out of Seven Barley Genotypes

The response of the tentative flavonoid was further elucidated in seven barley genotypes (Mundah, Keel, Vlamingh, Gairdner, Hindmarsh, Clipper, and Sahara) at five timepoints (8, 16, 24, 48, or 72 h) in control and saline conditions (250 mM NaCl) using LC-QToF-MS. The aim was to understand the differential expression of the detected tentative flavonoid in germinating seeds of a barley variety panel consisting of a small but representative set of six barley genotypes. **Figure S7** shows the differential change in the tentative flavonoid over time in seven genotypes. Hindmarsh, Clipper, and Gairdner showed no significant difference over time in both conditions. In Sahara, the highest increase was shown at 16 h under saline conditions. In Keel, a significant increase was observed at 72 h under saline conditions as compared to control seeds. A similar pattern was observed in Vlamingh where significant increase in the tentative flavonoid was seen at 72 h in salt treated seeds as compared to control seeds. In Mundah salt treated seeds, there was a decrease in the tentative flavonoid at 72 h. **Table S11** shows fold changes in signal response of the flavonoid for seven genotypes over time. Under saline conditions, among all genotypes, Vlamingh (+6.3-fold) and Keel (+14.04-fold) show the highest increase after 72 h salt stress (*p* < 0.001. However, in Mundah the highest increase was observed at 48 h (+9.0-fold) after salt stress with small decrease at 72 h (+8.9-fold) after salt stress.

### Elemental Concentrations in Seeds

**Figure 6:**
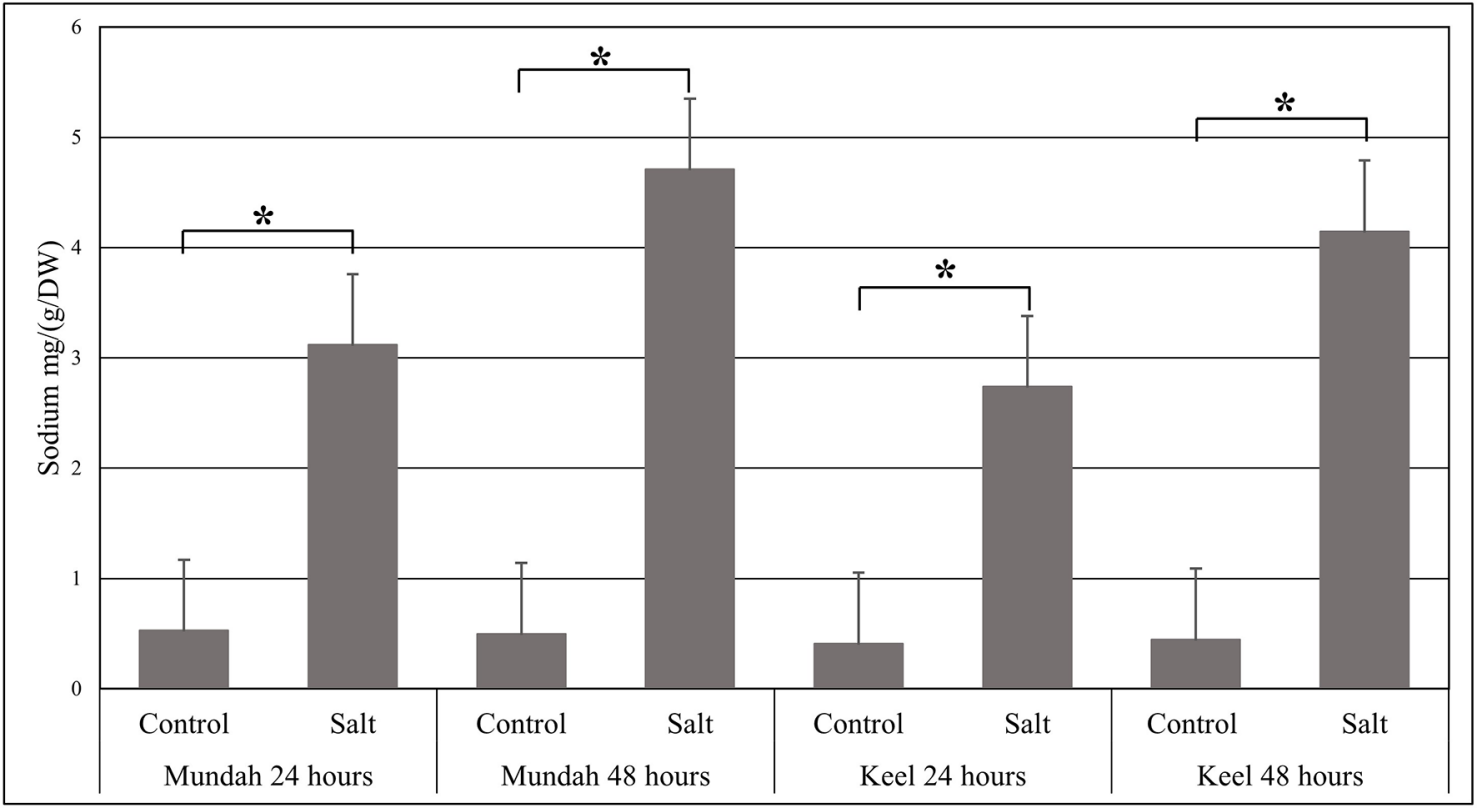
Change in the sodium concentrations identified by ICP-MS in Mundah and Keel seeds after 24 h and 48 h in control and salt treated conditions. Concentration of sodium mg g^−1^/dry weight (n = 4). Scales over the bar graph indicates SDs. Asterisks on the top of each bar highlight the significance levels between two treatments at individual timepoints (*P < 0.001).

The elemental composition of total barley seeds was analyzed to determine the effect of 24 and 48 h of salt stress (250 mM NaCl) on the content of eight macro- and micronutrients of both barley genotypes. **Table 3** summarizes the concentrations of elements in control and salt treated seeds and **Figure 6** shows the change in Na^+^ concentrations in both genotypes after 24 h in control and salt treated seeds. Of all the elements detected, only Na^+^ content was significantly altered (p < 0.001) after exposure to salt. In Mundah, Na^+^ concentrations were 0.5 ± 0.12 mg g^−1^ in control and 3.1 ± 0.48 mg g^−1^ after salt treatment for 24 h and 0.5 ± 0.06 mg g^−1^ in control and 4.7 ± 0.06 mg g^−1^ after salt treatment for 48 h. In Keel, Na^+^ concentrations were 0.4 ± 0.02 mg g^−1^ in control and 2.7 ± 0.21 mg g^−1^ after salt treatment for 24 h and 0.4 ± 0.06 mg g^−1^ in control and 4.2 ± 0.11 mg g^−1^ after salt treatment for 48 h. This indicated a significant increase in Na^+^ content in both Mundah (+9.4-fold) and Keel (+10.1-fold) after 48 h salt stress.

**Table 3:** ICP-MS of whole barley seed (Mundah and Keel) and its main tissue fractions (mg g^–^^1^ dry weight). Each tissue type was weighed and analyzed separately by ICP-MS.

Element	Mundah (24 h)		Mundah (48 h)		Keel (24 h)		Keel (48 h)	
	Control	Salt	Control	Salt	Control	Salt	Control	Salt
P 31	12.41 ± 1.69	12.89 ± 1.99	12.64 ± 2.89	14.29 ± 2.13	11.07 ± 1.9	12.17 ± 2.6	12.54 ± 4.43	13.34 ± 2.12
*Na 23	0.53 ± 0.12	3.12 ± 0.48	0.50 ± 0.06	4.71 ± 0.06	0.44 ± 0.02	2.74 ± 0.21	0.45 ± 0.06	4.16 ± 0.11
Mg 24	2.95 ± 0.22	2.72 ± 0.74	2.7 ± 0.61	2.94 ± 0.62	1.81 ± 0.5	2.21 ± 0.61	2.17 ± 0.95	2.36 ± 0.63
K 39	8.63 ± 1.24	8.23 ± 1.91	8.3 ± 2.04	8.05 ± 0.8	7.12 ± 1.18	7.01 ± 0.51	7.71 ± 2.49	7.58 ± 0.9
Ca 44	1.16 ± 0.08	0.76 ± 0.24	1.06 ± 0.3	0.85 ± 0.22	0.79 ± 0.22	0.7 ± 0.16	0.88 ± 0.39	0.79 ± 0.24
Mn 55	0.04 ± 0.01	0.04 ± 0.01	0.04 ± 0.01	0.04 ± 0.01	0.03 ± 0.01	0.03 ± 0.01	0.03 ± 0.01	0.03 ± 0.01
Fe-3 57	0.06 ± 0.01	0.06 ± 0.02	0.06 ± 0.02	0.06 ± 0.02	0.04 ± 0.01	0.05 ± 0.02	0.05 ± 0.03	0.06 ± 0.02
Zn 66	0.06 ± 0.01	0.06 ± 0.01	0.06 ± 0.02	0.07 ± 0.02	0.05 ± 0.01	0.06 ± 0.03	0.05 ± 0.03	0.07 ± 0.02

Data points represent as mean ± standard error, n = 3. Scale in mg g^−1^/DW.

*Values with significant differences (p < 0.001).

The K^+^ content in seeds decreased slightly after salt treatment but this was not statistically significant. None of the other six elements (P, Mg, Ca, Mn, Fe, and Zn) showed a statistically significant difference in seeds after salt stress as shown in **Table 3**. Since the overall concentration of most elements did not change during germination or in response to salt treatment we examined whether the treatments caused changes in the localization of the elements (S, P, K, and Cl) using µ-XRF analysis on seed sections from time point 24 and 48 h. In Mundah seeds, S accumulated in the entire seed irrespective of treatment. P was distributed in all seeds at moderate concentrations and was highly abundant in the aleurone layer. K was found in higher concentrations in control seeds than in salt treated seeds at all time points, with high abundance observed in the aleurone layer. These results complement the data obtained from ICP-MS. We were unable to observe Na^+^ due to instrumental limitations. However, Cl^−^ was used as an indirect measure of the Na^+^ distribution in seeds (Sarabia et al., 2018). Cl^−^ was highly abundant in salt treated seed sections with a uniform distribution correlating with the increased Na^+^ content as detected by ICP-MS (**Figure S8**). In Keel, S accumulated across the entire seed similarly to Mundah but was found to be more abundant in sections from salt-treated seeds obtained at 0 and 48 h post imbibition. P was also found to be highly accumulated in the aleurone layer of all sections and with lower abundance in the endosperm. K^+^ was seen abundantly in samples from the 0 h time point followed by a less intense distribution over time in both treatment. Cl^−^ was prominently abundant in salt treated seed sections correlating with the increased Na^+^ content detected by ICP-MS data (**Figure S9**).

## Discussion

In recent decades, salinization of agricultural land has resulted in lower crop productivity. Germination of seeds is directly affected by salinity either by the resulting osmotic stress or by toxic effects of the sodium and chloride ions themselves. In particular, salinity perturbs plant hormone balance (Khan and Rizvi, 1994) and reduces the utilization of seed reserves (Ahmad, 1992). In the past, metabolomics studies have mainly focused on the germination processes in rice and very little has been published on barley or on the effects of saline conditions on the germination process. In addition, these published studies used techniques that do not provide spatial information on metabolic processes. Gorzolka et al. (2016) previously studied the changes in metabolite profiles in barley seeds after 5 days of germination using MALDI-MSI, but changes in the metabolite profiles of barley seeds during the early stages of germination (0 to 72 h post imbibition) were not explored.

The process of uptake of water by a mature dry seed, which occurs in the initial hour of reintroduction of water to the dry seed, is triphasic (Bewley (1997) and results in the resumption of metabolic activity. These metabolic changes are extremely important to successful germination and, as such, it is vital to document and understand what changes occur (Bewley and Black, 1994; Bewley, 1997; Bewley, 2001). We first conducted a germination assay to establish the germination efficiency of seven barley genotypes under salinity stress. From these results, we selected two genotypes with contrasting germination phenology and salinity tolerance for this study: Mundah [early germinating and salinity tolerant, in agreement with Cao et al. (2017) and Keel late germinating and sensitive to salinity].

Following the germination test, ICP-MS and µ-XRF were combined to profile the levels and measure the spatial distribution of elements across six time points in the two selected barley genotypes (Mundah and Keel) under control and saline conditions. Munns and Tester (2008) reported that salt stress results in the accumulation of Na^+^ in plants and results in an adverse effect on K^+^ concentration which causes detrimental effects to plant growth and development. Various physiological studies have demonstrated that Na^+^ toxicity is caused due to the ability of Na^+^ to compete for K^+^ binding sites to disrupt K^+^ homeostasis (Volkov and Amtmann, 2006; Shabala and Cuin, 2008; Hasegawa, 2013). Hence, to avoid an ion homeostasis disorder under saline conditions, plant cells need to maintain a low Na^+^ concentration and a concurrent high K^+^ concentration in the cytosol, where enzymes for metabolism are located (Zhu, 2003; Pardo et al., 2006). In this study, we observed a substantial increase in Na^+^ content and a non-significant decrease in K^+^ content in germinating seeds of both genotypes under saline conditions.

It is apparent from this result that accumulation of excessive amounts of Na^+^ in germinating seeds are likely to be responsible for inhibition of germination under salt stress in Keel as compared to Mundah which was still able to germinate under these conditions. This view is supported by a previous report where increased accumulation of Na^+^ in wheat seeds under salinity stress inhibited the rate of germination (Begum et al., 1992). The K^+^/Na^+^ ratio decreased to greater extent in both Mundah and Keel after 48 h compared to 24 h salt stress. This result is in alignment with previously reported reduction of the K^+^/Na^+^ ratio in barley roots after salt stress (Shelden and Roessner, 2013). Salinity tolerance also depends on limiting Na^+^ accumulation and maintaining K^+^ content in the cytosol in order to achieve the preservation of ion homeostasis (Flowers et al., 1977; Hasegawa et al., 2000). Cl^−^ was observed to increase but no change in K^+^ was observed using µ-XRF after salt stress. Increased Cl^−^ distribution obtained from µ-XRF analysis is an indirect measure of Na^+^ as Cl^−^ is likely associated with Na^+^ as a counterion (**Figures S8–9**). The prevailing higher concentrations of these elements after salt stress are likely to be affecting metabolism and further impacting the observed levels of lipids and other metabolites in a germinating seed. It is worth noting that elemental composition in embryo region of both genotypes change after salt stress. It was observed that the embryo size in both conditions of two genotypes were increased under salt treatment. This is potentially due to role of osmoregulators that exhibit more abscisic acid (ABA) under salt stress. And increase in production of ABA induces the accumulation of more elements to be retained in those regions post-salt treatment (Sun et al., 2015).

Lipids are one of the major components stored exclusively in seed embryos and aleurone layer. They are vital and abundant cellular constituents responsible for cell membrane functionality as well as acting as an energy store to allow metabolism to continue during abiotic stress. The content of lipids in most cereals is relatively low (about 3%) compared to starch and protein. However, their contribution toward the nutritional value, as well as storage stability, of cereal-based food is important (Liu, 2011). Several studies have reported on the content and composition of lipids in whole grains (Price and Parsons, 1975; Welch, 1975; Zhou et al., 1998; Osman et al., 2000; Mehmood et al., 2008) without investigating spatial distributions. In this study, we investigated the effect of salt on the germination of two barley genotypes and on lipid composition and distribution using MALDI-MSI. High quality images taken at a 75 µm raster step size allowed observation of the spatial distribution of a high number of lipids and metabolites in barley seeds. The choice of matrix (DHB) also allowed tentative identification, and profiling, curation and annotation of a large number of lipids. This allowed observation of major lipid species showing differential spatial distributions across seed sections at all time points under control and saline conditions. Despite the higher proportion of lipid species in the embryo [20% lipids (Price and Parsons, 1979; Newman and Newman, 2008)], the largest number of lipids were detected in the endosperm region and not in the embryo. This could be due to the tissue type, as on-tissue extraction of ions is highly influenced by the properties of the tissue. For example, ion abundances can influence the adduct formation, and highly abundant, co-localized lipids or lipids bound to tissue structures can lead to ion suppression (Gorzolka et al., 2016). It could also be due to the thin tissue content of the embryo at these developmental stages which is highly hydrated (less dense) whereas the endosperm is still very dense.

Lipids play vital roles in providing structural integrity to cell membranes and are involved in signal transduction and membrane trafficking (Krauß and Haucke, 2007). In seeds, lipids serve as a reserve energy source and act as emulsifiers of fat substrates for lipases (Ory et al., 1967). Various environmental stresses including salinity have been shown to be responsible for changes in lipid metabolism and composition (Wang, 2002; Meijer and Munnik, 2003; Mansour, 2013; Natera et al., 2016; Sarabia et al., 2018; Yu et al., 2018). Lipids are rich in olefinic bonds which are susceptible to oxidative attack (Singh and Sinha, 2002; Parida and Das, 2005) from reactive oxygen species (ROSs) present in membranes during salinity stress (Esfandiari et al., 2007). The numbers of annotated species from the GP and ST classes decrease with increasing salinity in both genotypes. This is in line with findings by Wu et al. (2005) where analysis of changes in lipid composition following salt stress was studied in *Spartina patens*, a member of the Poaceae family. The decrease in the number of lipid species in Keel could also be due to peroxidation of lipids as mentioned by Pérez‐López et al. (2009).

Among the detected lipid classes in this study, phospholipids were major species of lipids that were tentatively annotated in both genotypes. This was indirectly supported by µ-XRF data where the distribution of P was found across the entire seed of both genotypes (**Figures S8–9**), correlating with regions where higher numbers of phospholipids were also observed using MSI. Presence of P in the aleurone layer from µ-XRF data also correlates with MALDI-MSI data where higher number of lipids were observed over time. Phospholipid metabolism is associated with a large number of biological molecules and plays a crucial role in various signal transduction pathways in higher plants. In response to various biotic and abiotic stresses (Munnik et al., 1998; Pappan and Wang, 1999), phospholipase D catalyzes the hydrolysis of structural phospholipids, PC and other phospholipids, resulting in the formation of PA. Ritchie and Gilroy (1998) reported that PA is an important mediator of abscisic acid (ABA) signal transduction in barley aleurone protoplasts. Levels of PA were observed to increase slightly with increasing salinity in both genotypes. Salinity stress dependent activation of PA could be due to activation of phospholipase D (PLD) that hydrolyses structural lipids such as PC and PE (Meijer et al., 2002). Increased levels of PA are also proposed to facilitate vesicular trafficking and to recruit target proteins to the membrane, influencing their activity (Testerink and Munnik, 2005; Wang, 2005; Roth, 2008).

High salt concentrations in the growing medium usually lead to an increase in the plasma membrane sterol content (López-Pérez et al., 2009). This increase facilitates hydrophobic interactions among the acyl chains, producing greater order in the membrane, and consequently, a more rigid membrane (Silva et al., 2011). In this study, the percentage of sterols decreased under salt stress in Mundah and Keel seeds. A seed usually has a pair of growing tips (apical meristems), that develop into stem (hypocotyl) and root (radicle). Thus, decrease in sterols in seeds of both genotypes could indicate a need for mobilizing lipids into meristematic tissue, providing nutrients during germination to support seedling growth during the early stages of development. It was also observed that sterol/phospholipid ratio was greatly affected after salt stress in Keel. Similar results were found in halophyte Brassicaceae species where a significant decrease in sterol lipids was observed after salt stress (Chalbi et al., 2015). However, the sterol/phospholipid ratio was unaffected by salt in Mundah which align with Wu et al. (2005) where the changes in lipid composition after salt stress in *Spartina patens* were studied.

Although phosphatidylcholine levels remained unchanged in Mundah seeds after salt treatment and increased in Keel after exposure to salinity, PC lipid species of 36:n was found at all timepoints in both genotypes under control and saline conditions. Similar results were found in the embryonic axis of *Camelina* seeds detected by MALDI-MSI under normal conditions (Horn et al., 2013; Horn and Chapman, 2014). Increased levels of PCs in response to high salinity was also found by Pical et al. (1999) in *Arabidopsis thaliana*. In plants, PCs are generally produced through a mixed cytidyl diphosphate-choline (CDP-Cho) pathways and methylation pathway (Tasseva et al., 2004). Tasseva et al. (2004) also suggested that enhanced synthesis of PCs is due to an accelerated CDP-Cho pathway. The accumulation of betaine in response to salt has been widely recognized in various plants. *In vitro* studies have proved the positive correlation between the accumulation of betaine and the acquisition of tolerance to salt in maize (*Zea mays*) (Rhodes et al., 1989) and barley (Strange, 1993; Kishitani et al., 1994). Hitz et al. (1981) demonstrated the use of phosphocholine for glycinebetaine synthesis in barley while Mcdonnell and Jones (1988) also proved that the choline required for oxidation of glycinebetaine is produced *via* turnover of PC. In our study, increased levels of PC over time could possibly result in the synthesis of glycinebetaine, helping seeds to cope under saline conditions. However, this particular mechanism needs further investigation.

High levels of Na^+^ also cause secondary responses in plants due to increased oxidative stress. Cellular damage from oxidative stress within plant cells (Apel and Hirt, 2004) induces production of reactive oxygen species (ROSs) (Jaleel et al., 2007; Ashraf, 2009). ROSs derived from molecular oxygen can accumulate in the plant cell and cause oxidative damage in cellular components, including proteins and lipids. To prevent the potential cytotoxic effects of ROSs, the stimulation of antioxidant systems can assist in plant protection from oxidative stress (Grace and Logan, 2000; Chutipaijit et al., 2009). Polyphenolic compounds such as flavonoids play an important role in stopping the propagation of oxidative chain reactions (Ksouri et al., 2007). Their synthesis is generally stimulated in response to various abiotic stresses including salinity (Zhou et al., 2018) and thus the biosynthesis of such compounds is generally stimulated in salt-exposed plants. Flavonoids are also potential inhibitors of the enzyme lipoxygenase, which converts polyunsaturated fatty acids to oxygen-containing derivatives (Nijveldt et al., 2001). These compounds accumulate in plant tissues protecting them from damaging effects of salt stress and inhibiting lipid peroxidation (Potapovich and Kostyuk, 2003). In our MSI results, a tentative flavonoid (*m/z* 365.102 [M+H^+^]) was found to be discriminant in Mundah and Keel embryos after exposure to salinity. Analysis using LC-MS/MS and MALDI-MS/MS confirmed the compound to be a flavonoid, tentatively annotated as Gaiconin F. Flavonoid profiling data from LC-QToF-MS supported our MALDI-MSI data (refer to **Figure S7** and **Table S11**). This is in alignment with Chutipaijit et al. (2009) who described a significant increase in total flavonoid content in salt stressed rice seedlings and Taïbi et al. (2016) where a significant increase in flavonoid content was seen in *Phaseolus vulgaris* under salt stress.

Under normal conditions, the quiescent embryo is able to germinate after imbibition and breaking dormancy (Mcdonald and Copeland, 2012). However, in salt stress conditions, the radicle has to avoid oxidative damage for successful germination. To detoxify or scavenge the severe effects of stress, the presence of antioxidants in embryos may play a crucial role in protecting the emerging radicle and help in a successful germination. This is also supported by Shirley (1998), who described the accumulation of flavonoids in the embryos of all plant species. Our observed detection of a tentative flavonoid in the embryos of Mundah and Keel supports the view of flavonoids playing potential roles in the protection of seeds under salt stress. Keel displayed a significantly delayed flavonoid response relative to Mundah as shown in the LC-MS data where highest X-fold increase in Keel was observed at 72 h. This depicts its lesser ability to deal with salt leading to poorer germination efficiency. However, in Mundah, flavonoid content was highest at 48 h displaying faster responses to salinity correlating with the higher germination efficiency. These results indicate a possible advantage for the Mundah genotype, making it a better germinator under salt stress. Gradual increase in the number of lipids detected over time from MALDI-MSI in Keel compared to Mundah under salt conditions also support our findings of Keel showing slow germination.

Analysis of the flavonoid profile of the five other barley genotypes showed varied and contrasting responses under salt stress over time. Vlamingh, which showed a germination efficiency between that of Keel and Mundah, also showed an increase in the Gancaonin F flavonoid under salt stress to a level between that of Keel and Mundah. These observations point to Gancaonin F providing some advantage to specific genotypes in the response to salt stress through possible antioxidant scavenging of ROS species and protection of the vulnerable embryo during germination. The other four genotypes showed a variety of differing patterns in Gancaonin F profiles. There are a multitude of possible response mechanisms that the germinating seed is able to employ and the varying responses observed are most likely due to the wide range in genetic diversity of the selected barley varieties.

## Conclusions

This study investigated the changes in levels and distribution of metabolites and elements in germinating seeds of Mundah and Keel, two barley genotypes that have contrasting germination rates in response to salt stress. To compare and contrast the lipid and metabolite profiles of these varieties during the early stages of seed germination under control and saline conditions, several orthogonal approaches were combined using MALDI-MSI, elemental composition analysis using ICP-MS, spatial distribution analysis using µ-XRF spectrometry and confirmation of compounds using LC-QToF-MS. Use of discriminative MALDI-MSI analysis as an exploratory and qualitative technique allowed visualization of larger lipid differences within different seeds structures as well as detection of a tentative flavonoid that showed discriminate behaviour between the two genotypes at different time points. Further analysis across genotypes to determine the roles of flavonoids in barley germination under salt stress conditions is required. However, these initial results indicate flavonoid as a strong candidate metabolite biomarker for detecting salinity tolerant varieties.

## Data Availability

This manuscript contains previously unpublished data. The raw data supporting the conclusions of this manuscript will be made available by the authors, without undue reservation, to any qualified researcher.

## Author Contributions

SG carried out the experiments, data analysis and interpretation of the results. SG wrote the manuscript with support from BB, SN, PS, CH, UR. LCMS was conducted and analyzed by SG and TR. ICP-MS was conducted and analyzed by SG and DC. µ-XRF imaging was conducted by BB. Both BB and UR supervised the project. All authors provided critical feedback, helped shape the research and authorized the final manuscript.

## Funding

This project and UR were funded through an Australian Research Council (ARC) Future Fellowship program (Grant Number FT130100326).

## Conflict of Interest Statement

The authors declare that the research was conducted in the absence of any commercial or financial relationships that could be construed as a potential conflict of interest.
