# Supplementary material for: Spatio-Temporal Metabolite and Elemental Profiling of Salt Stressed Barley Seeds During Initial Stages of Germination by MALDI-MSI and µ-XRF Spectrometry
Source: Front Plant Sci. 2019 Sep 25;10:1139. doi: 10.3389/fpls.2019.01139 (PMC6774343; doi:10.3389/fpls.2019.01139)
Supplement: Supplementary file 1 [file DataSheet_1.pdf]

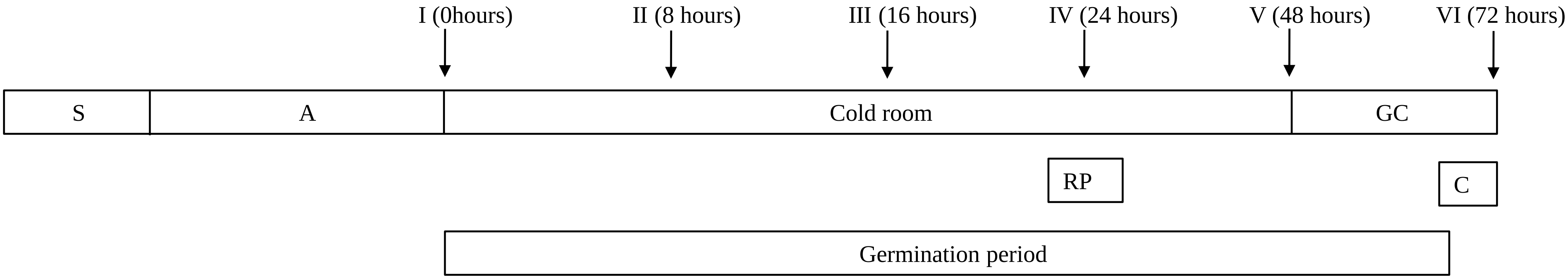

Supplementary Figure 1. Timepoints for barley genotypes germinated for the collection of samples. Arrows indicate timepoints for collection of samples. I: 0 hours, II: 8 hours, III: 16 hours, IV: 24 hours, V: 48 hours, VI: 72 hours post aeration. S: sterilization of seeds, A: overnight aeration, GC: growth cabinet (dark, constant 17°C), RP: radicle protrusion, C: coleoptile emergence, Germination period: starts from emergence of radicle (visible germination) until final sample collection (72hours).

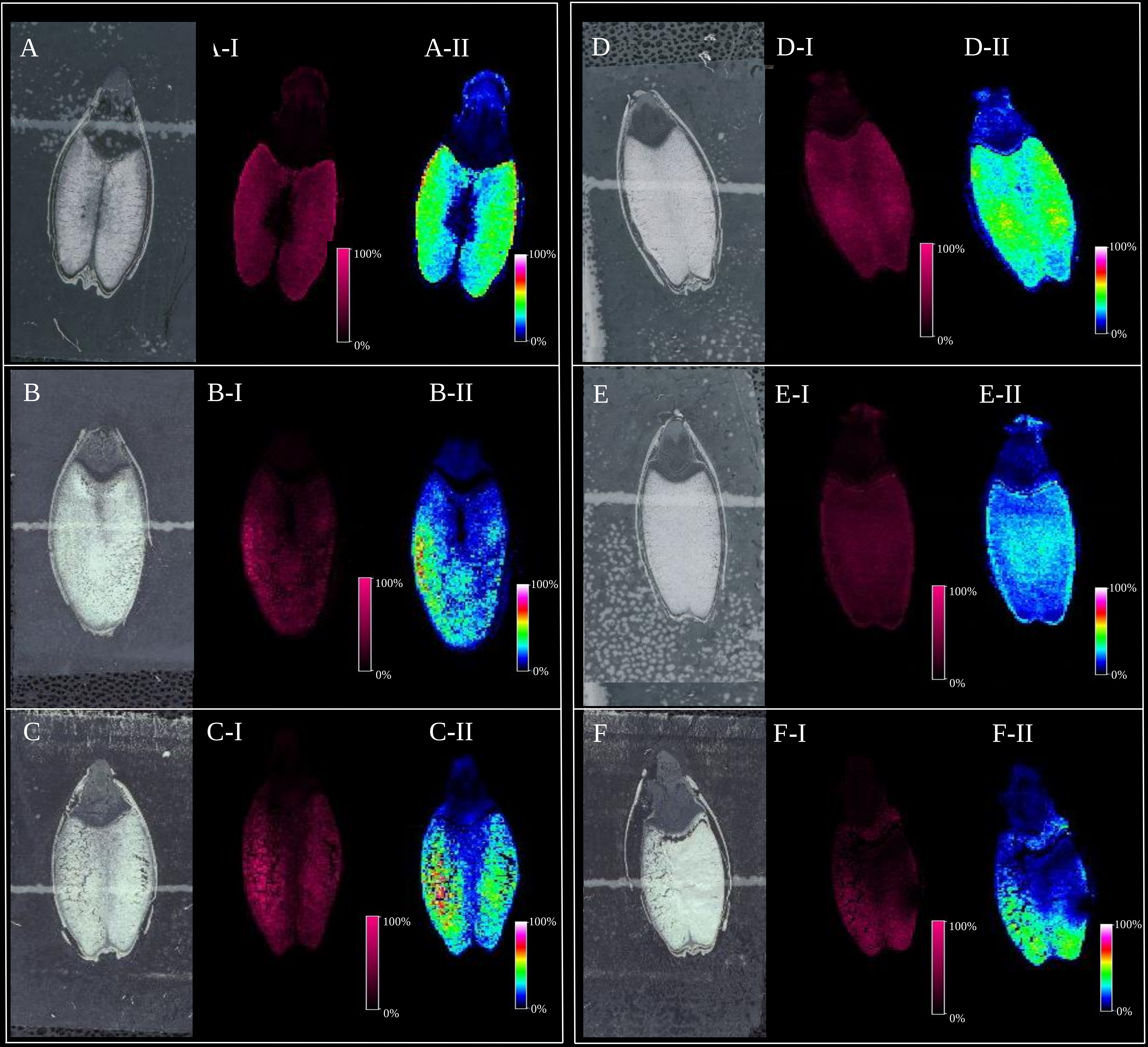

Supplementary Figure 2. Tissue distribution of *m/z* 796.52 PC(34:2) observed in three independent replicates at 16 hours in Mundah control and salt treated seeds. A, B, C: scanned image of Mundah control seed replicates 1,2 and 3; A-I, B-I, C-I: single colour filter for *m/z* 796.52 $[M+K]^+$ showing the intensity scale of 0-100%. A-II, B-II, C-II: colour gradient filter to visualize the distribution of *m/z* 796.52 $[M+K]^+$ with intensity scale of 0-100%. D, E, F: scanned image of Mundah salt treated seed replicates 1, 2 and 3; D-I, E-I, F-I: single colour filter for *m/z* 796.52 $[M+K]^+$ showing the intensity scale of 100%. D-II, E-II, F-II: colour gradient filter to visualize the distribution of *m/z* 796.52 $[M+K]^+$ with intensity scale of 100%.

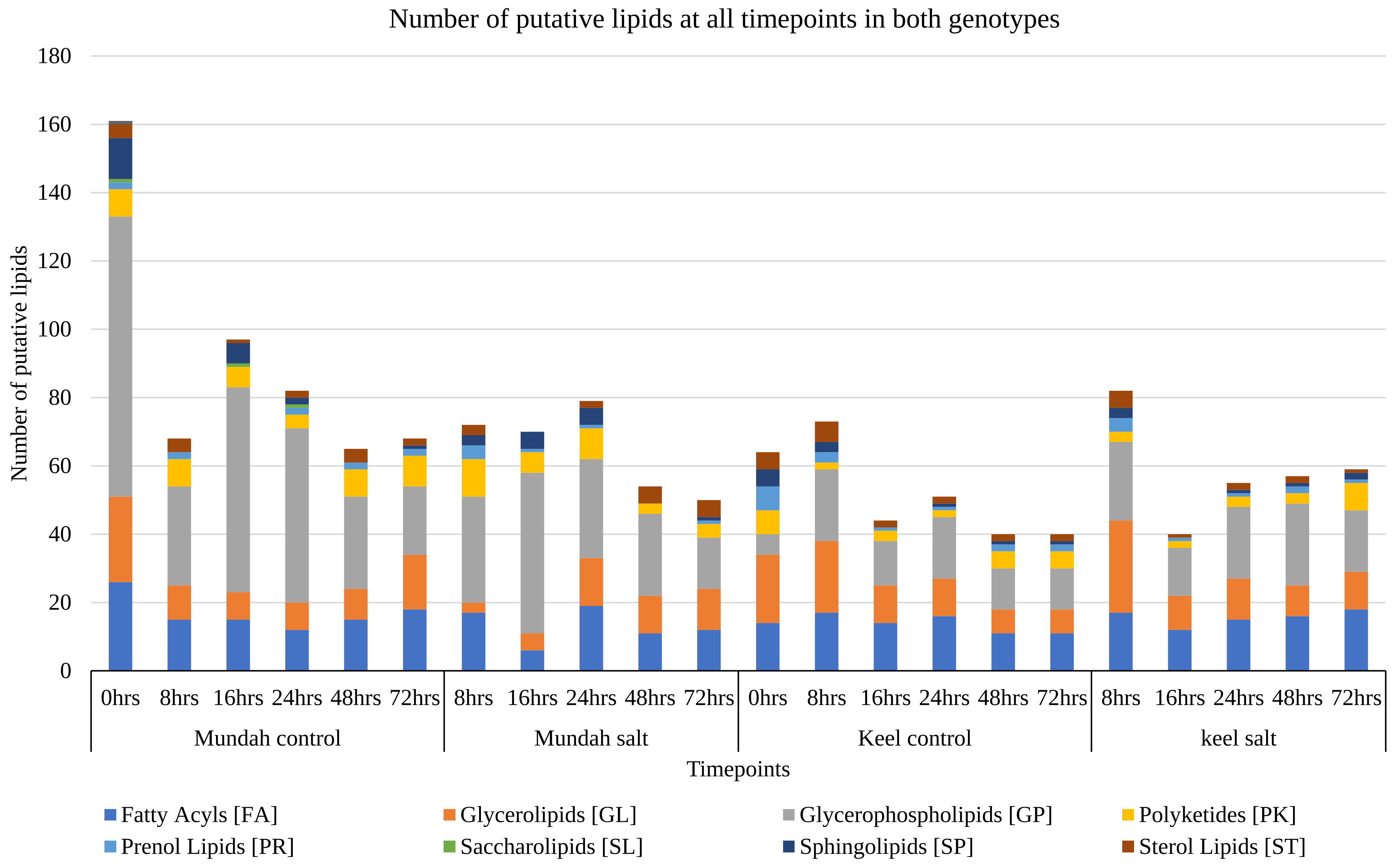

Supplementary Figure 3. Distribution of total lipid classes at all time points in cv. Mundah and cv. Keel germinating seed under control and salt-treated conditions. The X-axis shows the six time points for control and 5 time points for salt treated samples. The Y-axis indicates the number of lipids putatively identified at each time point. The putative lipid identities shown in this graph were established using an accurate mass precursor ion search (<5 ppm) of the LIPID MAPS database. Note: These identities are not collapsed into single ID and hence there is repetition of masses at different time points.

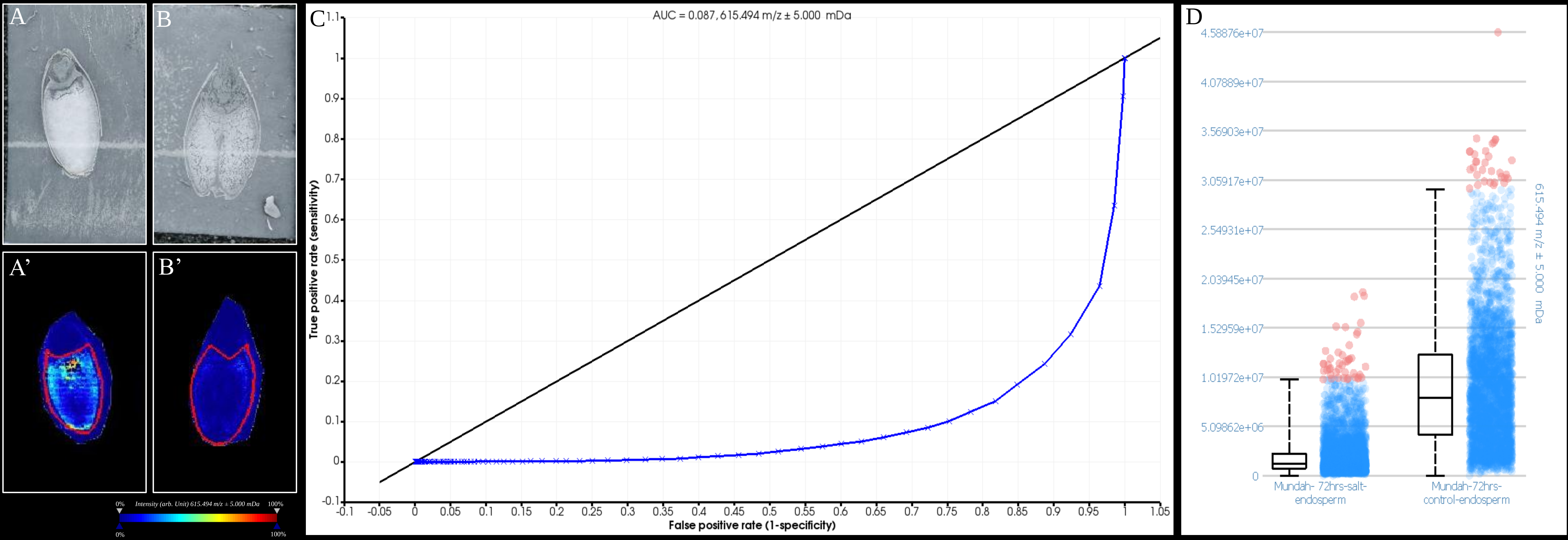

Supplementary Figure 4. ROC plot for *m/z* 615.494 $[M+Na]^+$ DAG(34:2) from Mundah endosperm. AUC was 0.087 showing high intensity of this ion in control seed of Mundah at 72 hours. A: Mundah control 72 hours seed scanned image, B: Mundah salt treated 72 hours seed scanned image, A': Mundah control 72 hours showing DG(34:2) in the endosperm region, B': Mundah endosperm region for salt treated seed at 72 hours. C: ROC plot for *m/z* 615.494 showing true positive rate higher than 0.8. D: Intensity box plot chart for two selected endospermic regions. Red lines indicate sub regions created in SCiLS Lab. Here, red bordered regions are the endosperms. Intensity scale was set to 0-100%.

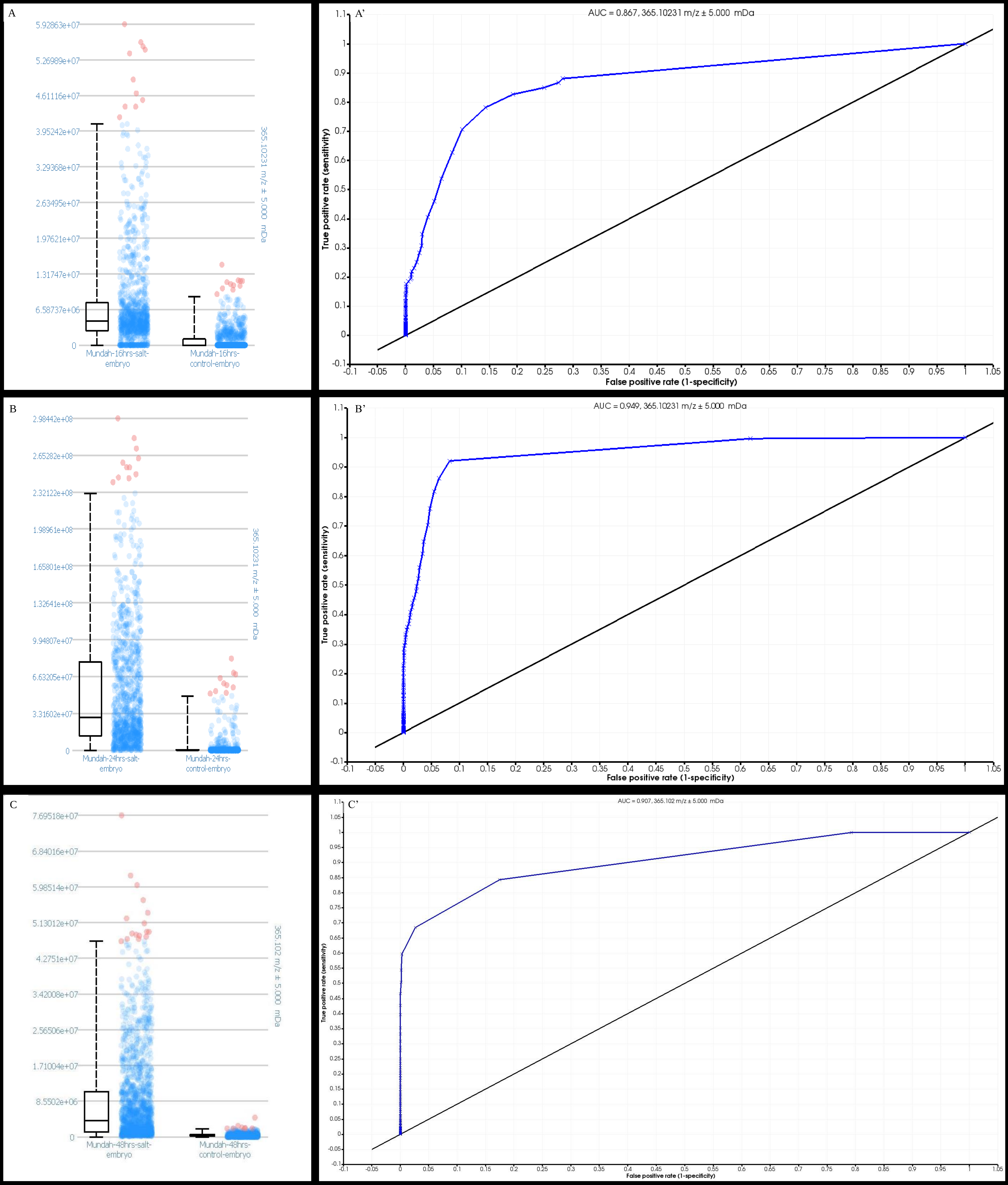

Supplementary Figure 5a. The intensity box plots and ROC plots for a putative flavonoid (*m/z* 365.102) observed in Mundah at 16, 24 and 48 hours. A, B, C: The intensity box plots at 16, 24 and 48 hours. The box part contains a rectangle divided by a horizontal line, that represents a median intensity. Lower and upper bounds of the box represent the total number of spectra with intensities below these lines in one quarter and three quarters respectively. The cloud part of the plot shows scattering of spectra of a given region by intensity of a given *m/z* interval. Blue dots represent the spectra in which intensities of a given *m/z* interval are between the lower and upper quantiles whereas red dots represent the spectra whose intensities are outliers. A', B', C': ROC plots at 16, 24 and 48 hours showing the discrimination capabilities of given *m/z* for two (control and salt) regions with AUC values of 0.867, 0.952 and 0.907 respectively.

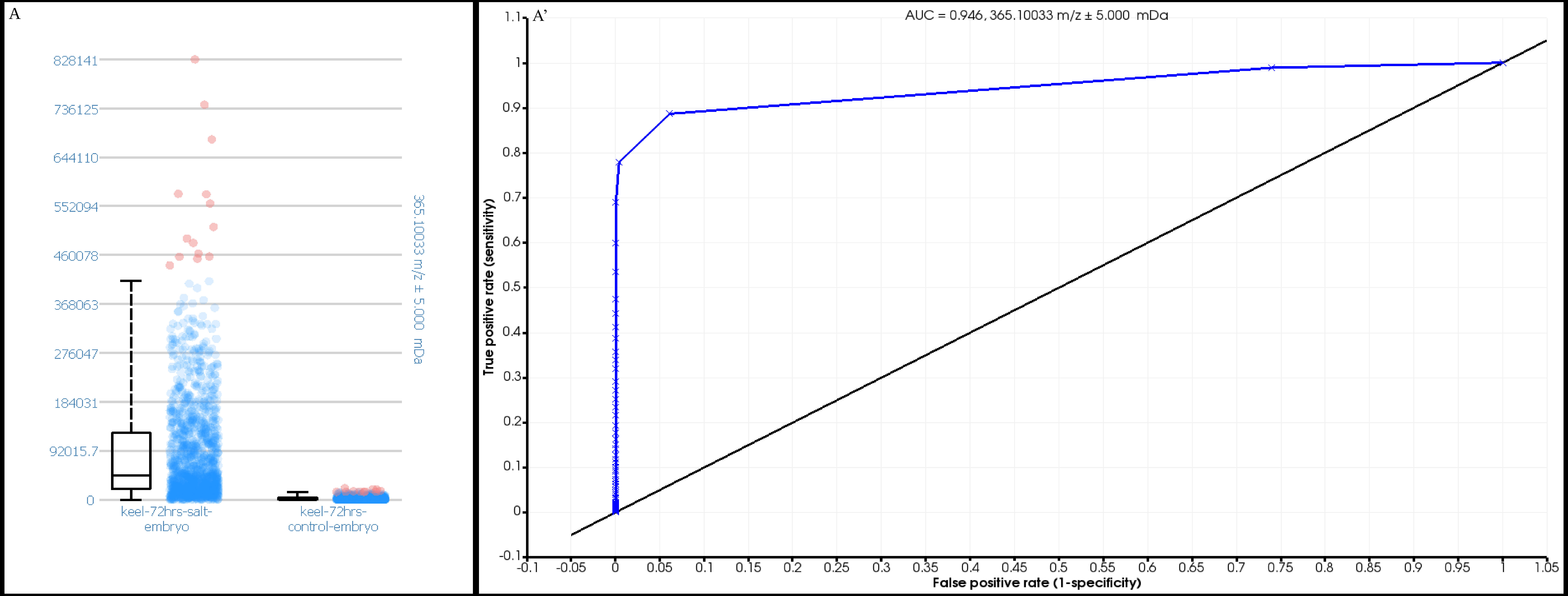

Supplementary Figure 5b. The intensity box plots and ROC plots (AUC value of 0.953) for a putative flavonoid (*m/z* 364.0947) observed in Keel at 72 hours.

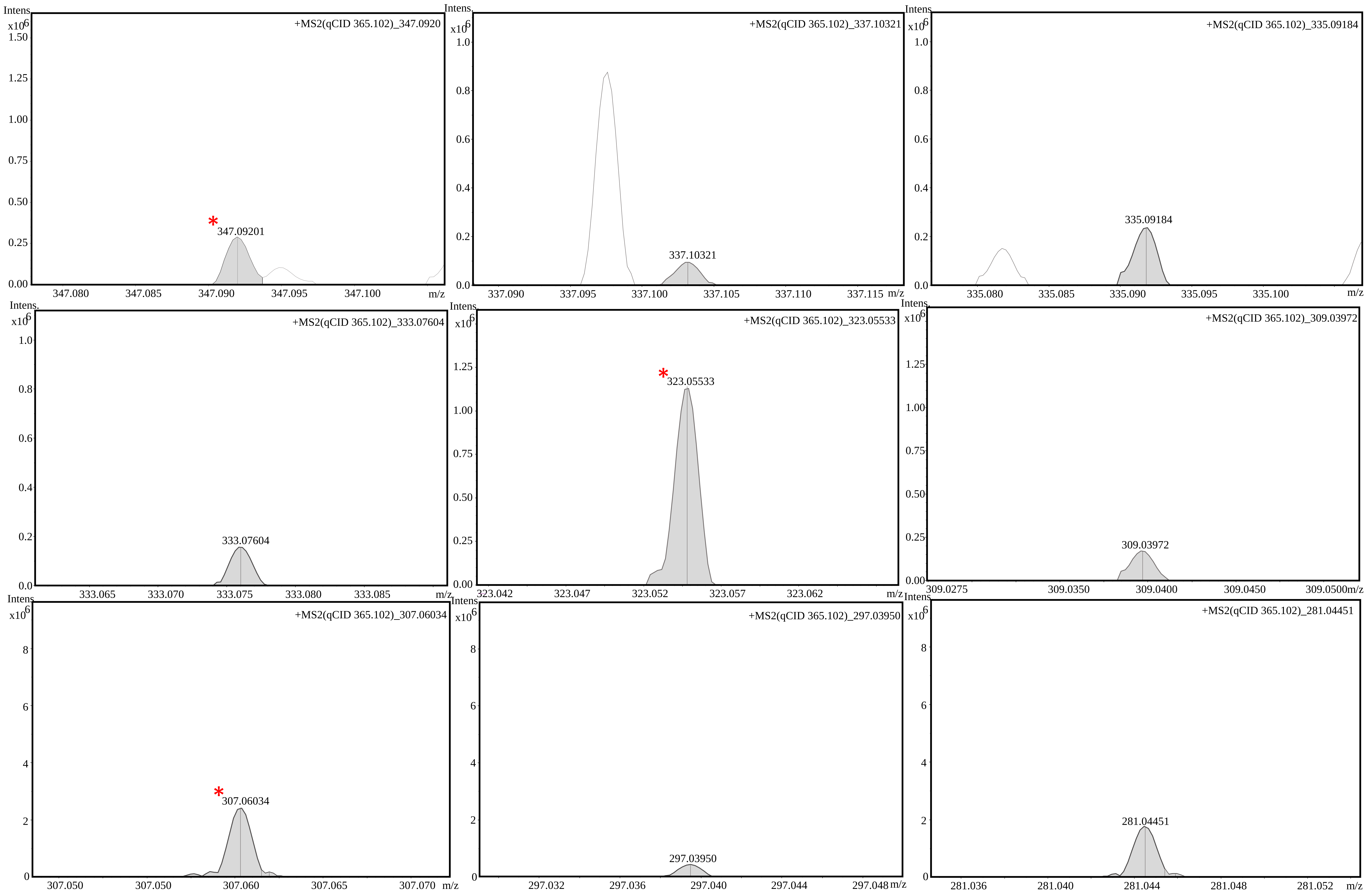

Supplementary Figure 6. MS/MS fragments of precursor ion with *m/z* 365.102 $[M+H]^+$. Nine product ions were detected with *m/z* 347.0920, *m/z* 337.1032, *m/z* 335.0918, *m/z* 333.0760, *m/z* 323.0553, *m/z* 309.0397, *m/z* 307.0603, *m/z* 297.0395 and *m/z* 281.0445. Asterisks represent fragments matched with LC-MS/MS analysis shown in figure 5.

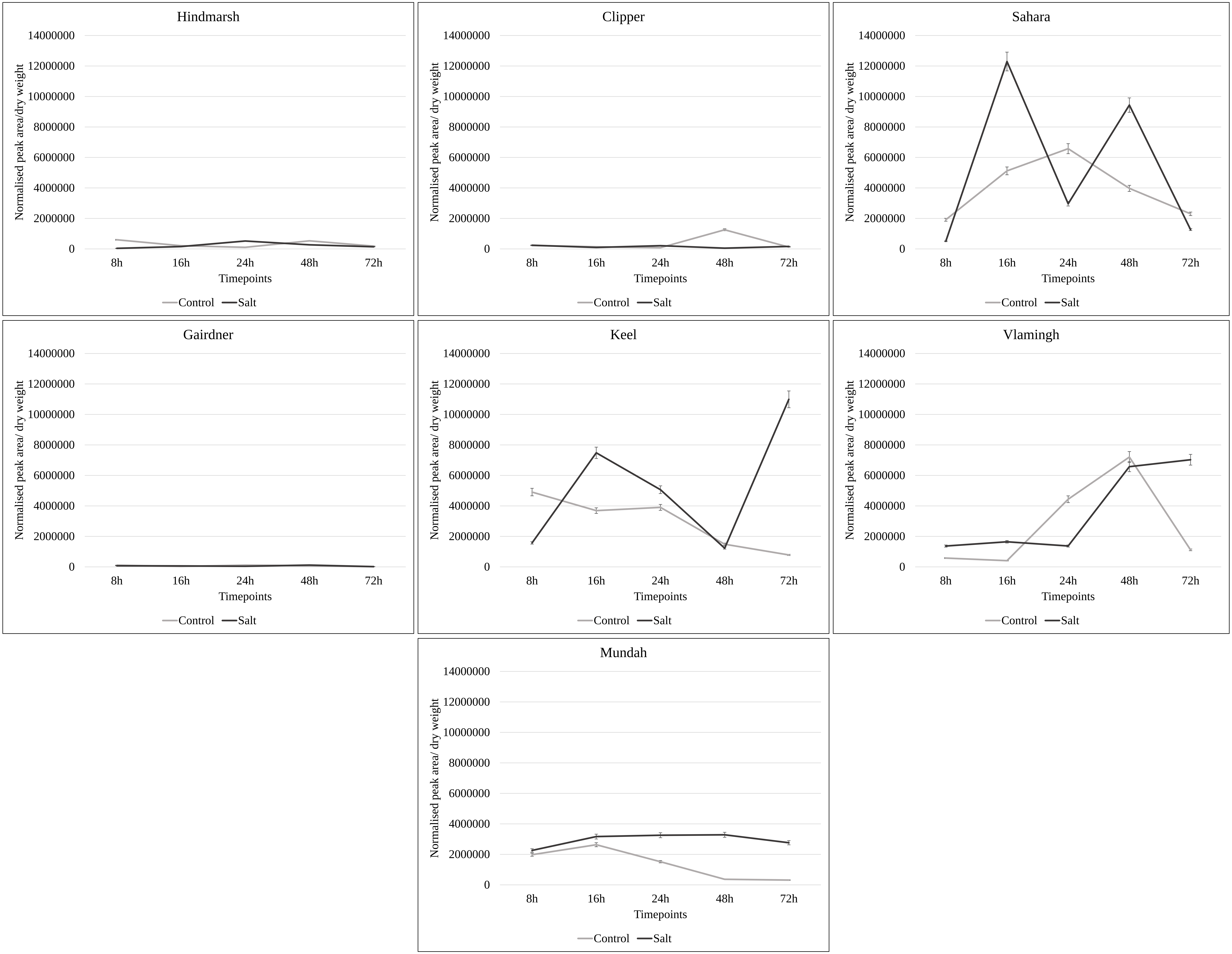

Supplementary Figure 7. Line graph of normalised log responses of *m/z* 365.102 at 6 different timepoints (8 hours, 16 hours, 24 hours, 48 hours and 72 hours) in seven genotypes under control and saline conditions measured using LC-QToF-MS. X-axis indicates timepoints and Y-axis indicate normalized log response.

0hours	24hours		48hours	
	0mM NaCl	250mM NaCl	0mM NaCl	250mM NaCl

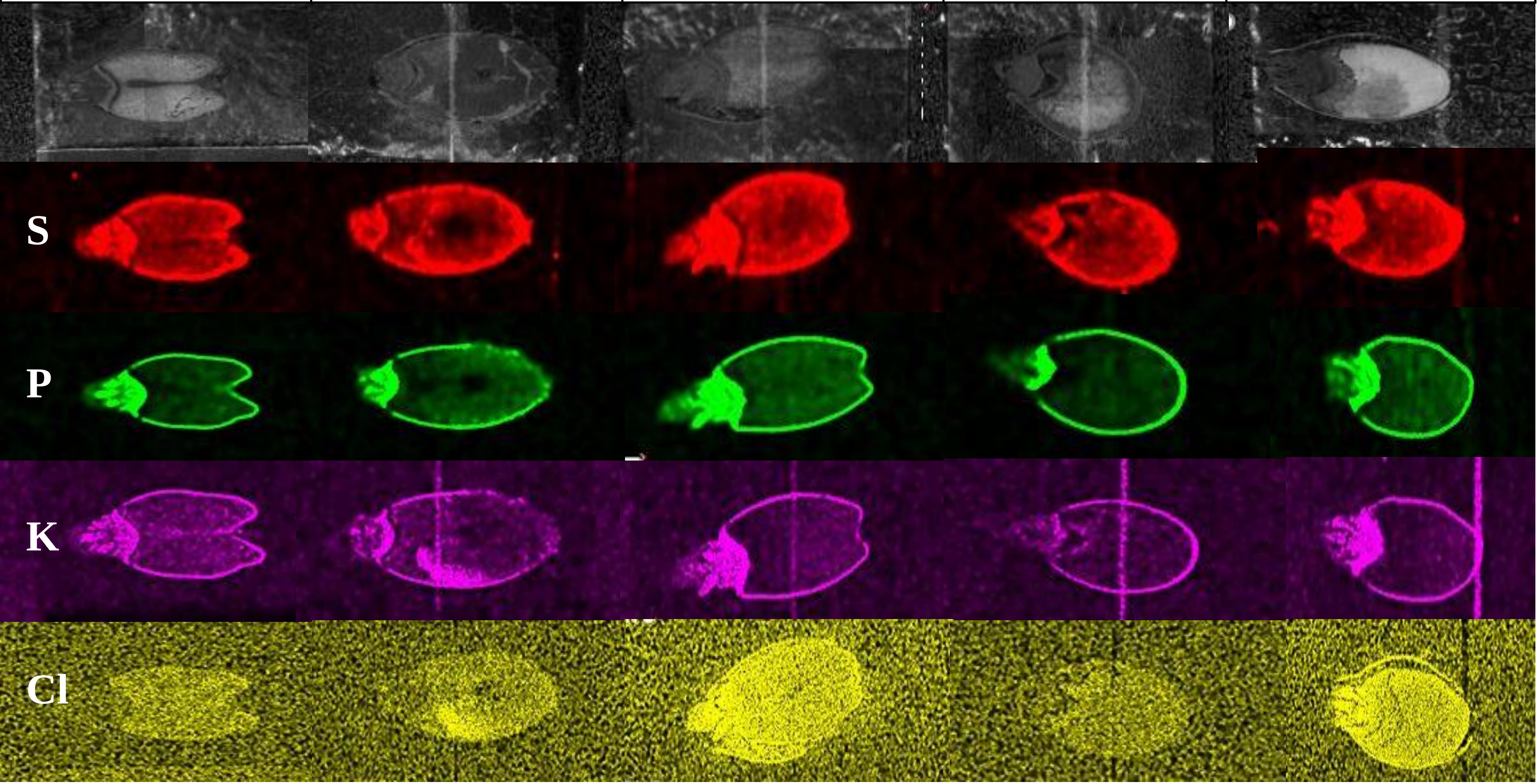

Supplementary Figure 8. The elemental maps of S, P, K and Cl in germinating barley seeds of Mundah under control and saline conditions. Each image indicates the relative distribution of the specific element captured at 10 ms/pixel.

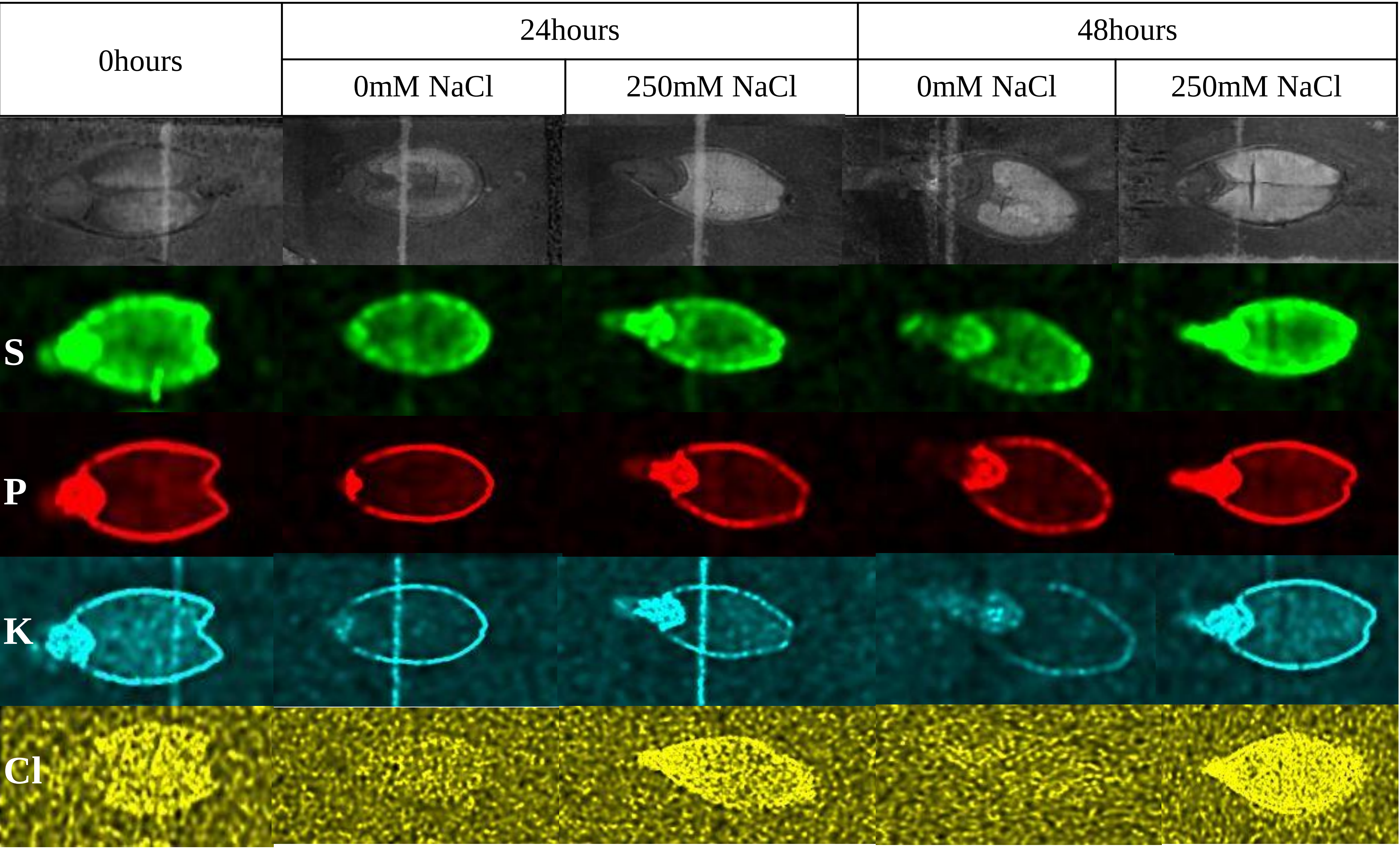

Supplementary Figure 9. The elemental maps of S, P, K and Cl in germinating barley seeds of Keel under control and saline conditions. Each image indicates the relative distribution of the specific element captured at 10 ms/pixel.
